# Uncovering missing pieces: duplication and deletion history of arrestins in deuterostomes

**DOI:** 10.1186/s12862-017-1001-4

**Published:** 2017-07-06

**Authors:** Henrike Indrischek, Sonja J. Prohaska, Vsevolod V. Gurevich, Eugenia V. Gurevich, Peter F. Stadler

**Affiliations:** 1 Computational EvoDevo Group, Department of Computer Science, Universität Leipzig, Härtelstraße 16–18, Leipzig, D-04107 Germany; 2 Bioinformatics Group, Department of Computer Science, Universität Leipzig, Härtelstraße 16–18, Leipzig, D-04107 Germany; 3 Interdisciplinary Center for Bioinformatics, Universität Leipzig, Härtelstraße 16–18, Leipzig, D-04107 Germany; 4 Department of Pharmacology, Vanderbilt University, 2200 Pierce Ave, Nashville, TN 37232 USA; 5 Max Planck Institute for Mathematics in the Sciences, Inselstraße 22, Leipzig, D-04103 Germany; 6 Fraunhofer Institute for Cell Therapy and Immunology, Perlickstraße 1, Leipzig, D-04103 Germany; 7 Department of Theoretical Chemistry, University of Vienna, Währinger Straße 17, Vienna, A-1090 Austria; 8 Center for non-coding RNA in Technology and Health, Grønegårdsvej 3, Frederiksberg C, DK-1870 Denmark; 9 Santa Fe Institute, 1399 Hyde Park Rd., Santa Fe, NM 87501 USA

**Keywords:** Arrestin, Signaling, Gene duplication, Evolution, Receptor specificity, Retrogene

## Abstract

**Background:**

The cytosolic arrestin proteins mediate desensitization of activated G protein-coupled receptors (GPCRs) via competition with G proteins for the active phosphorylated receptors. Arrestins in active, including receptor-bound, conformation are also transducers of signaling. Therefore, this protein family is an attractive therapeutic target. The signaling outcome is believed to be a result of structural and sequence-dependent interactions of arrestins with GPCRs and other protein partners. Here we elucidated the detailed evolution of arrestins in deuterostomes.

**Results:**

Identity and number of arrestin paralogs were determined searching deuterostome genomes and gene expression data. In contrast to standard gene prediction methods, our strategy first detects exons situated on different scaffolds and then solves the problem of assigning them to the correct gene. This increases both the completeness and the accuracy of the annotation in comparison to conventional database search strategies applied by the community. The employed strategy enabled us to map in detail the duplication- and deletion history of arrestin paralogs including tandem duplications, pseudogenizations and the formation of retrogenes. The two rounds of whole genome duplications in the vertebrate stem lineage gave rise to four arrestin paralogs. Surprisingly, visual arrestin *ARR3* was lost in the mammalian clades Afrotheria and Xenarthra. Duplications in specific clades, on the other hand, must have given rise to new paralogs that show signatures of diversification in functional elements important for receptor binding and phosphate sensing.

**Conclusion:**

The current study traces the functional evolution of deuterostome arrestins in unprecedented detail. Based on a precise re-annotation of the exon-intron structure at nucleotide resolution, we infer the gain and loss of paralogs and patterns of conservation, co-variation and selection.

**Electronic supplementary material:**

The online version of this article (doi:10.1186/s12862-017-1001-4) contains supplementary material, which is available to authorized users.

## Background

Arrestins are cytosolic proteins with a molecular weight of about 40-45 kDa involved in the regulation of cell signaling. The binding of arrestins to activated and phosphorylated G protein-coupled receptors (GPCRs) blocks the inter-helical cavity of active GPCR, thereby precluding its coupling to cognate G proteins [1, 2]. Thus, arrestins contribute to the fast and precise shut-off of GPCR signaling via G proteins. In particular, arrestin binding is indispensable for a high temporal resolution in vision [3, 4]. Beyond their “arresting”-function that gave the protein family its name, diverse other biological functions of arrestins have been described in the last two decades. Among them are the scaffolding, subcellular localization, and regulation of kinases, phosphatases and ubiquitin ligases, G protein independent signaling and GPCR trafficking (for review see [5, 6]). In recent years, considerable efforts were made towards the design of arrestins that modulate GPCR signaling and facilitate biased signaling [7].

**Fig. 1:**
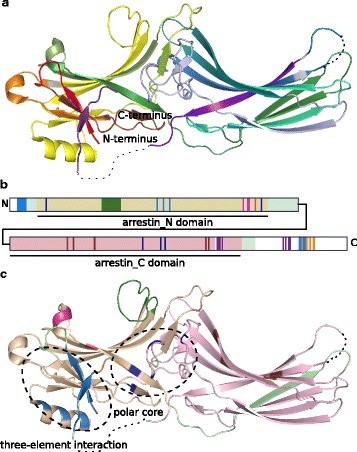
Functional elements of arrestins. **a** – Crystal structure of bovine *ARRB1* colored according to the conserved exon-borders in vertebrates (rainbow coloring from exon 2 - *red* to exon 16 - *dark violet*). Exons 1, 15 as well as parts of exons 13, 14 and 16 are missing in the crystal structure (shown as dotted lines if not situated on the N- or C-terminus). Amino acids whose codons are split among two exons are shown in *grey* [112]. **b** – Schematic, linear representation of bovine *ARRB1* with important functional elements shown in bright colors (*orange* - AP-2 binding site, *light blue* - three-element interaction, *dark blue* - polar core, green - finger loop, brown - high affinity IP6 binding site, *pink* - low affinity IP6 binding site, *red* - phosphate sensor, *purple* - clathrin binding sites). Arrestins encode two key domains, the arrestin_N domain (wheat) and the arrestin_C domain (*light pink*). Other regions that are present in the crystal structure are shown in light green, while sequence parts missing therein are shown in white. **c** – Functional elements depicted in B are mapped to the crystal structure of bovine *ARRB1*. The clathrin binding sites are missing in the crystal structure as they are situated on exons 13 and 15. PDB: 1G4R [55]. Crystal structure images were created with Pymol 1.8.4.0 Open-Source [113]

Arrestin proteins consist of two domains each with the *β*-sandwich at its core, the arrestin_N and arrestin_C domain. The domains are connected by a highly flexible linker region. The N domain contains the only *α*-helix (Fig. 1 a). Arrestin proteins belong to the arrestin clan and were named *β*-arrestins by [8] or true arrestins by [2, 9]. Below, we will refer to this group of proteins as arrestins although there are additional members in the clan that share the anti-parallel *β*-sandwich fold and are involved in cellular trafficking. These are the arrestin-domain containing proteins and a set of families that are rather distantly related to arrestins with maximal 10% sequence identity [8]. These distant relatives encompass the VPS26 family (including DSCR3) and RGP1 that are represented in human (*Homo sapiens*), as well as fungal arrestin-related trafficking adapters, amoebal arrestin domain-containing proteins and the Spo0M family in bacteria and archaea [8, 10].

Arrestins have been found in Choanoflagellata, Filasterea and Metazoa, which all belong to Holozoa [11]. Within Metazoa, arrestins are found in both deuterostomes and protostomes [8, 9, 11]. In contrast to the rest of the arrestin clan, the sequences of arrestins are highly conserved [5]. Mammalian arrestins were studied extensively in the past [12–14]. There are four paralogs, functionally divided into the visual and non-visual group, each composed of two members. The visual arrestins, arrestin-1 (formerly known as rod arrestin) and arrestin-4 (formerly known as cone arrestin or X-arrestin) are encoded by the genes *SAG* and *ARR3*, respectively. The non-visual arrestins, arrestin-2 and arrestin-3 (also known as b-arrestin1 and b-arrestin2), are encoded in humans by the genes *ARRB1* and *ARRB2*. Both functional groups are clearly monophyletic. Visual arrestins exhibit a much higher evolutionary rate than non-visual arrestins [15, 16].

GPCRs engage the concave side of arrestins [17–19]. Receptor binding leads to the reorganization of arrestin’s polar core and three-element interaction inducing a conformational change and resulting in the release of arrestin’s C-terminus [17, 20, 21]. The C-terminus of non-visual arrestins harbors binding sites for AP-2 and clathrin (Fig. 1 b, c) [1].

Arrestin-1 is the prevalent form in mouse cones, suggesting that it can bind to cone pigments [22]. Arrestin-1 is well known for binding to rhodopsin with high specificity, preferring it over other GPCRs [19, 23]. In contrast, binding specificity of arrestin-4 is ensured by its co-expression with cone opsins in cone photoreceptors, as in vitro arrestin-4 binds non-visual GPCRs fairly well [24]. In contrast, non-visual arrestins are expressed in all cell types and have a broad receptor specificity recognizing several hundred different GPCRs.

Individual arrestins from non-mammalian vertebrates have been cloned for functional studies. Among them are visual arrestins from frogs [25–27], salamander [13] and gecko [28]. Phylogenetic analyses support 1:1 orthology with their human counterparts. [29] reported co-expression of two distinct arrestin-1 genes, termed *SAGa* and *SAGb*, in rods of medaka (*Oryzias latipes*) and [3] identified two zebrafish paralogs (*Danio rerio*) for each visual arrestin ortholog in human, as well as two zebrafish paralogs for arrestin-3. They concluded that three additional arrestin genes originated from the teleost-specific whole genome duplication event (3R-WGD). [15] reported the expression of a visual and a non-visual arrestin in arctic lamprey’s pineal organ (*Lethenteron camtschaticum*). [30] showed that the vase tunicate (*Ciona intestinalis*), has only a single arrestin with functional features of both visual and non-visual subtypes. This suggests that the divergence of visual and non-visual arrestins is indeed associated with the vertebrate-specific whole genome duplications (2R-WGD). A comprehensive phylogenetic analysis to test this hypothesis, however, still has been missing.

While the cloning of individual arrestins led to the discovery of unexpected duplications and subfunctionalizations, the evolutionary history of arrestins has not been studied systematically. The information on arrestin homologs presently available covers only a very limited range of species [8] and an incomplete set of paralogs for most species investigated [9]. The objective of this study was to systematically investigate the duplication and deletion history of arrestins in deuterostomes. Sequence and exon-intron structure conservation are evaluated to gain insight into possible functional changes of the less studied members of the protein family and to elucidate nature’s repertoire of signaling interfaces relating to arrestins.

## Results

**Fig. 2:**
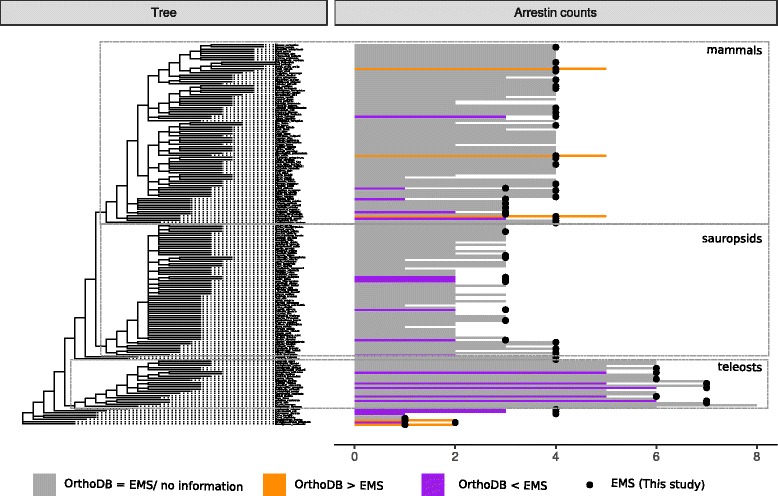
Number of deuterostome arrestin paralogs resulting from the application of the ExonMatchSolver (EMS) pipeline and manual curation in comparison with the OrthoDB database. Higher and lower paralog counts were obtained by genome mining in combination with manual curation for 20 species (*purple*) and five species (*orange*), respectively, as compared to the OrthoDB. The paralog counts and annotations obtained with the EMS approach and that are based on an expert opinion, are assumed to be correct. OrthoDB overpredicted sequences due to mis-assembly (*Sus scrofa*), inclusion of a pseudogene (*Monodelphis domestica*), a naming mistake (*Homo sapiens*), included two additional sequences without any further reference (*Branchiostoma floridae*, *Saccoglossus kowalevskii*). Arrestins correspond to the OrthoDB group EOG091G05M2

We were working with two data sets that resolve arrestin phylogeny on two levels. In a database analysis, we placed arrestins in a wider evolutionary context (first subsection), while in a second analysis we focused on a narrower set of sequences covering only deuterostome arrestins (all other subsections). The interest in the detection of positive selection and co-variation requires a complete collection of paralogs per genome, a highly accurate annotation of the exon-intron structure on nucleotide level and transfer of the functional annotation between homologs. For this purpose, we needed carefully reconstructed coding sequences of the individual family members even when situated on genome fragments (see Methods). This level of accuracy is currently not provided by databases for non-model organisms. This has been a limitation to previous studies on arrestin evolution. We applied the ExonMatchSolver (EMS) pipeline and manually curated the annotation of deuterostome arrestins. We demonstrate that in comparison to a coarse database analysis, the exon-intron structure focused homology search is in fact a more successful strategy to trace the details of arrestin evolution. For example, considering paralog counts, OrthoDB under- and overpredicted the number of paralogs in 20 and five of 57 species, respectively. In general, we found paralogs that are missing from OrthoDB (Fig. 2). OrthoDB overpredicted sequences due to mis-assembly (in pig, *Sus scrofa*), inclusion of a pseudogene (in opossum, *Monodelphis domestica*), a naming mistake (in human), and included two additional sequences without any further reference (in lancelet, *Branchiostoma floridae* and acorn worm, *Saccoglossus kowalevskii*). We added five species critical to resolve the arrestin genealogy that were not included in OrthoDB (*Lytechinus variegatus*, *Patiria miniata*, *Leucoraja erinacea*, arctic lamprey and *Orycteropus afer afer*). The updated annotation is in general more complete than the respective database entries and represent a fundamental improvement in regard to the annotation of splice sites, short and terminal exons. We argue that our approach demonstrates how detailed curation can change and improve the detailed duplication and deletion history of an individual gene. The updated arrestin annotation represents one of the very rare instances of a highly curated set of paralogous genes and thus is ideal for evaluation of gene annotation tools and orthology prediction tools.

### Placing deuterostome arrestins in a wider evolutionary context

To obtain an updated overview of the evolution of proteins that harbor an arrestin_N and arrestin_C domain, we queried UniProtKB and OrthoDB in a jackhmmer search with profile Hidden Markov Models (pHMMs) built from the four human arrestin full-length sequences. We found very remote homology to the scaffolding proteins DSCR3 and VPS26B, that contain a Vps26 domain, as reported previously in [8, 10]. The following domains are members of the Arrestin N-like clan (CL0135) in Pfam 31.0, which corresponds to the arrestin clan described in the literature: arrestin_C, arrestin_N, Spo0M, Vps26 and transcends this classification by inclusion of the domains LDB19 and Bul1_N (both restricted to Fungi). Restricting our search to a homology level, where the arrestin_N domain and arrestin_C domain can be detected with their respective Pfam HMMs (PF00339, PF02752), results in a set of ten members in human in accordance with [8] (*ARRDC1-5*, *TXNIP*, *SAG*, *ARRB1*, *ARRB2*, *ARR3*). We refer to this group as the arrestin fold family. These homologs form four orthologous groups supported by phylogenetic inference with both, full-length and single domain sequences (arrestins, *ARRDC1*, *ARRDC2/ARRDC3/ARRDC4/TXNIP*, *ARRDC5*, Additional file 1: Figure S1, Additional file 2). The monophyly of each group, arrestins and the *ARRDC2-4/TXNIP*, is further supported by the strict conservation of their exon-intron structure within the respective groups in humans (arrestins: 13-16 exons, see Fig. 10, *ARRDC2-4/TXNIP*: 8 exons). *ARRDC1* shares three exon-intron boundaries with the *ARRDC2-4/TXNIP* group supporting *ARRDC1* as the closest outgroup to *ARRDC2-4/TXNIP*, while *ARRDC5* shares the two existing exon-intron boundaries with both, *ARRDC1* and *ARRDC2-4/TXNIP*. The arrestin and *ARRDC2-4/TXNIP* groups expanded at the base of vertebrates with generally lower paralog numbers in non-vertebrate deuterostomes and protostomes (Additional file 1: Figures S2 and S4). While the majority of vertebrate arrestin fold family members belongs to one of these four orthology groups (OrthoDB-IDs: EOG091G0B0Y, EOG091G07XG, EOG091G0CVZ, EOG091G05M2), more diversity is seen in protostomes with numerous lineage- or clade-specific extensions (Additional file 1: Figure S2 A). Striking lineage-specific extensions occurred e.g. in Caenorhabditis (nematodes) and Polypedilum (flies), that posses up to 30 arrestin homologs as described by [8, 11]. The emergence of the arrestin, *ARRDC1* and *ARRDC2-4/TXNIP* groups predates the split of protostomes and deuterostomes, while the *ARRDC5* group is amniota-specific (Additional file 1: Figure S2 A). At least two of the four surveyed metazoan species outside of Bilateria additionally possess three orthology groups that do not have representatives in human (Additional file 1: Figure S2 A). To determine the existence of arrestin homologs in even deeper branching clades, we considered the results of the scan of Pfam arrestin domain models (PF00339, PF02752) and the full-length human arrestins against UniProtKB, which covers more species than OrthoDB (Additional file 1: Figure S3). 79% of all our hits against the UniProtKB database with the full-length query contain at least one arrestin_N and one arrestin_C domain covering the clades of Metazoa, Fungi, Amoebozoa, Alveolata and Stramenophiles with at least three species representatives of each of these clades (Additional file 1: Figures S3 and S5).

We additionally detected hits in the following clades with one representative each: bacteria (*Sorangium cellulosum*), virus (*Canarypox virus*) and Chlorophyta (*Chlorella variabilis*). Our results confirm the absence of arrestin fold proteins in Embryophyta and their low abundance in Chlorophyta described by [11]. Our phylogenetic inference also confirms that the arrestin fold protein in *Canarypox virus* probably originated from horizontal gene transfer of a vertebrate member of the *ARRDC2-4/TXNIP* group (Additional file 1: Figure S1) [10]. Arrestins clearly form a monophyletic group within the group of arrestin fold proteins, which expanded in deuterostomes to give rise to the four paralogs seen in humans (Fig. 2).

### Emergence of the four vertebrate arrestin paralogs by whole genome duplications (2R-WGD)

The arrestin sequences retrieved from the genomes of jawed vertebrates (the updated annotation) fall into four well separated orthology groups, each of which contains one of the four human arrestins (Additional files 3 and 4). Phylogenetic trees of the gene family, furthermore, show that the visual arrestins, *SAG* and *ARR3*, form a well supported monophyletic group. Disregarding *ARRB2* of lampreys, the same applies to the non-visual arrestins, *ARRB1* and *ARRB2*. The split of non-visual arrestins and *ARR0* is well supported in the Bayesian tree and the Maximum likelihood tree with selected sequences (see ML tree with basal arrestins in Additional file 1: Figure S7 and Bayesian tree in Additional file 4), while this split is not well supported in the ML tree including all curated arrestin sequences. In order to check that this tree topology is not the result of convergent evolution of visual arrestins, we removed the parts of the sequence that are known to mediate receptor binding [2, 17, 19, 23, 31–33]. The truncated alignment still produces the same tree topology (Additional file 5).

**Fig. 3:**
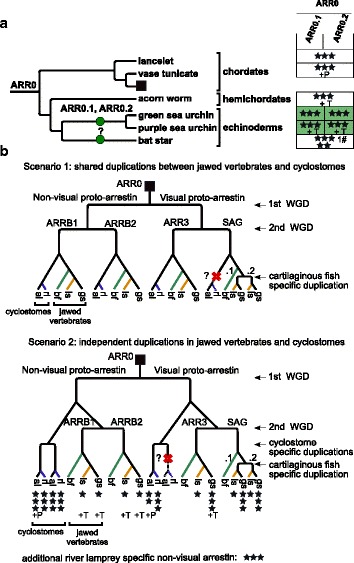
Duplication and deletion of arrestin paralogs within basal deuterostomes. **a** - Species tree of basal deuterostomes with mapped duplication events of arrestins (*dots*). **b** - Schematic arrestin gene tree for vertebrates (*square* in A). A cross indicates a gene loss. New gene names are given above the dot or branch. The gene loss/ duplication pattern was simplified for bony fish (bf), see Fig. 6, Fig. 8 and Additional file 1: Figure S13 for details. The completeness of arrestin annotations in the respective genomes is depicted with three stars indicating 0-3 missing exons, two stars 4-8 missing exons, one star more than 8 missing exons and dash (-) that no gene fragments were detected. Additional support from other omics-data for cartilaginous fishes and jawless fishes and from experimentally validated genbank entries is indicated by the following abbreviations: T - transcriptome evidence, P - protein evidence. The hash (#) indicates the number of frame shift mutations contained in the exon annotation. Note that the order of cyclostome-specific and cartilaginous fish-specific duplications in relation to each other was chosen arbitrarily. An additional non-visual arrestin detected in the germline genome of river lamprey was not included in the scenario (see Additional file 1: Appendix 1). Phylogenetic trees were created with Treegraph 2.0.54 [114]

The scenario best supported by the data is the existence of one visual and one non-visual proto-arrestin derived from a single arrestin, referred to hereafter as *ARR0* (Fig. 3). *ARR0* subsequently gave rise to two arrestins each (Fig. 3 b). All investigated non-vertebrate arrestins cluster together in a well-supported subtree. *ARR0* is most similar to the non-visual vertebrate arrestins, especially to *ARRB1* (average identity of all *ARR0* to human *ARRB1* 61.9%).

**Fig. 4:**
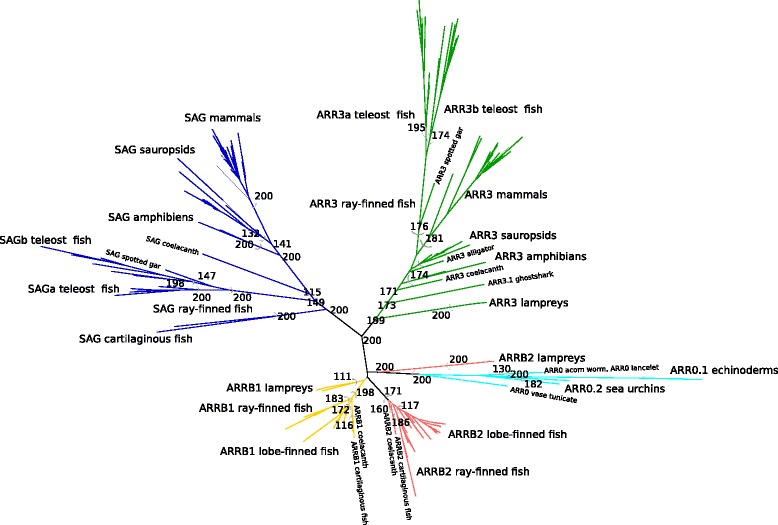
Maximum likelihood tree of arrestins. The tree was constructed from an amino acid alignment of deuterostome arrestins using PhyML (model JTT+I+G with *α* 1.04, 0.05% of invariable sites and 200 bootstraps). The different monophyletic and well-supported orthology groups are highlighted in different colors. Bootstrap support values from 50...100% are shown for the labeled monophyletic groups. The phylogenetic tree was visualized with Dendroscope 3.5.7 [115]

In order to pinpoint the exact timing of the divergence of the four vertebrate arrestins, we focused on arrestins in available genomes of river lamprey (*Petromyzon marinus*) and arctic lamprey. Cyclostomes, including lampreys, are the sister clade of the jawed vertebrates (Gnathostomes). The pattern of arrestin distribution in lampreys is heterogeneous with different numbers of paralogs retrieved from the germline and somatic genome of river lamprey. However, the germline genomes of both lamprey species harbor at least one visual and two non-visual arrestins that are clearly 1:1 orthologs (Fig. 4, Additional file 3). A third, complete non-visual arrestin is encoded in the germline genome of river lamprey (see Additional file 1: Appendix 1 for details about arrestins in lampreys). One group of lamprey non-visual arrestins (*ARRB2* lampreys) clusters with *ARR0* with high support, while the other forms a monophyletic group with vertebrate *ARRB1*, albeit with low support (Fig. 4, Additional file 4). The visual arrestin from arctic lamprey clusters together with vertebrate *ARR3* with high support. The position of the putative lamprey *ARRB1* and *ARR3* within the tree is in agreement with a shared 2R-WGD. However, the exact timing of the emergence of the four arrestin paralogs and thus the exact timing of the first and second round of the 2R-WGD cannot be resolved unambiguously with the available data, see Fig. 3 b for two possible tree scenarios. We return to this issue in the discussion.

### Tandem duplication of *ARR0* in sea urchins

**Fig. 5:**
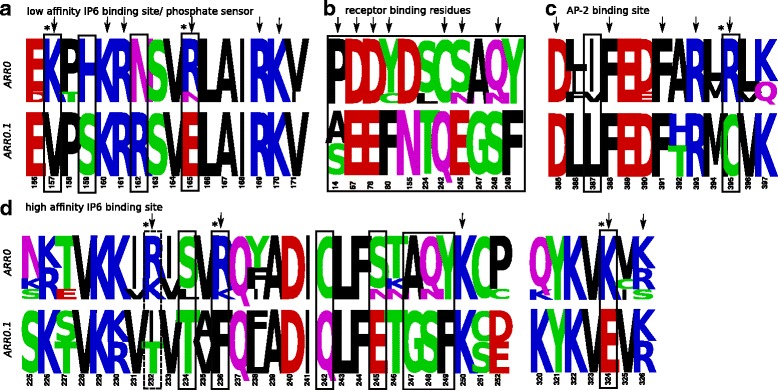
Specificity determining positions discriminating between sea urchin *ARR0.1* and *ARR0*s including *ARR0.2* from sea urchins. Amino acid frequency logos are shown for *ARR0* and *ARR0.1* of sea urchins ordered by functionality of motifs known from studies in vertebrate arrestins (**a**–**d**). Positions that are known to directly confer the respective functionality are marked by arrows. Some mutations change the charge of the respective residue (marked with *). Positions identified by SDP analysis are highlighted by black boxes. As receptor specificity is mediated by a rather big interface, only the SDPs are shown that are known to be involved in receptor binding and their direct neighbors. An additional position that shows differences in both groups (manually identified) and is associated with the respective function is highlighted by a dotted box. The numbering of the positions refers to bovine *ARRB1*. See the following references: [2] (pos. 14), [32] (pos. 67, 78, 80, 82 in finger loop region), [17] (pos. 154, 233), [19] (pos. 242), [23] (pos. 245, 247, 248, 249) for receptor binding residues, [34] (pos. 157, 160, 161, 165, 232, 236, 250, 324, 326) for IP6 binding residues, [19] (pos. 165, 169) for phosphate sensing and [35] (pos. 385, 388, 391, 393, 395) for AP-2 binding residues. Results are also summarized in Additional file 6. The figure was created with Weblogo [116]

Most non-vertebrate deuterostome genomes encode a single *ARR0* gene (Fig. 3 a). The most notable exceptions are three echinoderms. The sea urchins *Strongylocentrotus purpuratus* and *Lytechinus variegatus* possess two paralogous *ARR0* genes with a mean sequence identity of 61%. They are located about 110 kb apart indicating that they are the result of a tandem duplication. The *ARR0.1* genes show an accelerated substitution rate in comparison to *ARR0.2* as indicated by long branch lengths within the phylogenetic tree. *ARR0.1* of both sea urchins carries specificity determining positions (SDP) that are distinct from homologous positions in all other investigated *ARR0*s. These differences include charge reversing substitutions at positions known to be important for phosphate sensing [19], inositol-6-phosphate (IP6) binding [34] and AP-2 binding [35] (Fig. 5 a, c, d, see Additional file 6). Furthermore, receptor binding residues are different (Fig. 5 b). After the duplication and before speciation of green and purple sea urchin, different fractions of sites evolved under positive selection in *ARR0.1* and *ARR0.2*, 15% and 5%, respectively (see Additional file 6). Before speciation of green and purple sea urchin, positions involved in or neighboring to receptor binding sites as well as to IP6 binding residues are positively selected in the *ARR0.1* branch (Table 1). Furthermore, we find sequences in the bat star *Patiria miniata* suggesting the presence of two *ARR0* genes, despite their near identity (exonic nucleotide sequences are 98.7% identical, intronic nucleotide sequences are 89% identical). Clearly, these two copies are the result of a very recent duplication independent of the duplication event that generated the much older paralogs in the sea urchins.

**Table 1:** Positively selected residues detected with the BEB method

Foreground branch	BEB sites	Homologous position in cow paralog	Function known from homologs	SDP?
*ARR0.1* sea urchins	S76	N83	Second neighboring to receptor binding residue	x
	E95	E102	-	x
	K116	K157	Low affinity IP6 binding site	x
	N121	N162	Neighboring to low affinity IP6 binding site	x
	N184	N225	Second neighboring to receptor binding residue	-
	C201	C242	Receptor binding	x
	N296	N382	Second neighboring to clathrin binding site	-
*ARR0.2* sea urchins	K82	P89	Neighboring to PxxP motif	-
*SAG.1* ghost shark	K2	K2	-	na
	V106	P134	Neighboring to receptor binding residue	na
	N114	R171	Phosphate sensor	na
	T128	G185	Neighboring to PxxP motif	na
	N160	G217	-	na
	H205	E262	Receptor binding	na
	Q248	N305	Second neighboring to polar core	na
	V277	T334	Second neighboring to high affinity IP6 binding site	na
	S27	G27	Second neighboring to receptor binding residue	na
*SAGb* teleost	V14	V35	Second neighboring to polar core	x
	R126	W194	Receptor binding	-
*SAGb* Acanthopterygii	P72	P93	Neighboring to PxxP motif	na
	A112	A180	Neighboring to PxxP motif	na
	D142	S210	-	na
*ARR3b* Euteleosteomorpha	Y42	M55	Neighboring to mu2 adaptin binding site	x
	C150	F254	Neighboring to receptor binding residue	x

The branch-site model of the PAML package was used to identify sites under positive selection in the specified foreground branch. The position in column two refers to the position within the group alignment, while the homologous position in cow serves as a reference. The position in *ARR0* is given in respect to *ARRB1* in cow. The function assignment is based on literature review. See Additional file 6 for further details. Positions that were also identified as specificity determining position (SDP), are marked by a cross. SDP were not determined for all subgroups as indicated by “na”

### Tandem duplication of *SAG* in cartilaginous fishes

The jawed vertebrates are divided into two major subgroups, the bony fish (including reptiles, birds, and mammals) and the cartilaginous fish comprising the chimaeras, sharks, and rays. *SAG* is tandem-duplicated in the ghost shark genome, the only available chimaera genome (*Callorhinchus milii*). The two copies, *SAG.1* and *SAG.2*, are located about 8 kb apart on opposite strands. With the help of the EMS pipeline and additional manual curation, we also found support for a second *SAG* gene in the draft assembly of the genome of the little skate, *Leucoraja erinacea* (see Additional file 1: Appendix 2 for details on annotation of arrestins in cartilaginous fish). Therefore, the tandem duplication of *SAG* occurred before the split of chimaeras and sharks between 413-473 mya (Fig. 3 b). The protein sequences of arrestin-1.1 and arrestin-1.2 of ghost shark have an identity of 51% and 55%, respectively, to the single arrestin-1 of spotted gar. About 13% of sites are under positive selection in ghost shark *SAG.1* (Table 1, see Additional file 6). Among these are two residues involved or directly neighboring to a receptor binding residue. The basic residue R171 is conserved among *SAG*, with only a few exceptions. However, it is replaced by an acidic asparagine in ghost shark’s arrestin-1.1., probably impairing its function as a phosphate sensor.

### Increase of arrestin number in ray-finned fish as a consequence of 3R-WGD

**Fig. 6:**
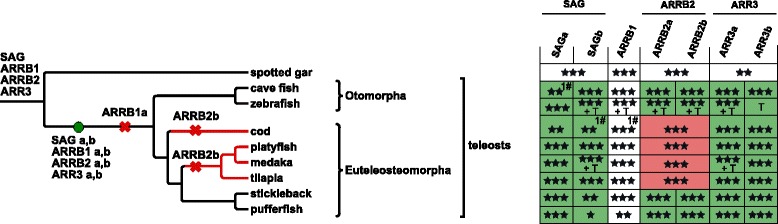
Duplication and deletion of arrestin paralogs within ray-finned fish. The teleost whole genome duplication (WGD) increases the number of arrestin paralogs within the two clades Otomorpha and Euteleosteomorpha. The two resulting copies of each paralog (*SAG, ARRB1, ARRB2, ARR3*) are depicted as a and b. Zebrafish *ARR3b* was annotated in GRCz10 as the gene was missing in the originally investigated genome version Zv9. The species tree was created based on [117] using Treegraph 2.0.54 [114]. Crosses depict gene losses. See caption of Fig. 3 for additional description of symbols

The bony fish are formed by the class of lobe-finned and ray-finned fish, with the majority of living representatives of the latter falling into the infraclass of teleosts (Fig. 6). The genome of spotted gar (*Lepisosteus oculatus*), a ray-finned fish outside the teleosts, encodes four arrestin paralogs, while all investigated teleosts have six or seven arrestin genes (Fig. 6), confirming and extending the results of [29] and [3]. The increased number of paralogs is explained by the teleost-specific round of genome duplications (3R-WGD) that happened between 230-315 mya (Additional file 1: Figure S7). 3R-WGD potentially resulted in eight arrestin paralogs [3]. We hypothesize that one copy of *ARRB1* was lost already before the divergence of Otomorpha and Euteleosteomorpha during the initial 85 million years (max.) after the 3R-WGD (Fig. 6, Additional file 1: Figure S7) [36]. The other three pairs of copies are retained in the ancestor of the eight investigated teleosts. The ancestral *ARRB2b* evolved under neutral evolution and was lost independently along two different branches of Euteleosteomorpha (Fig. 6), while the majority (80%) of *ARRB2a* sites evolved under purifying selection directly after duplication. *ARRB2a* and *ARRB2b* of Otomorpha are overall very similar (average of 90% identity), while *ARRB2b* of stickleback (*Gasterosteus aculeatus*) and pufferfish (*Takifugu rubripes*) are a lot more divergent from *ARRB2a* of the same species (average 79.3% identity). Supervised multiple correspondence analysis (MCA) shows each of these two sequences as a separate cluster that is also clearly distinct from the group of all other *ARRB2* in teleosts. This observation strongly suggests a change in function. Differences identified by manual inspection concern residues binding to IP6 (K161Q) [34], the phosphate sensor R166 (mutated to Q) [19], AP-2 binding residues (R395G) [35] as well as residues that mediate receptor specificity (e.g. P253Q/S) [19] (see Additional file 6).

**Fig. 7:**
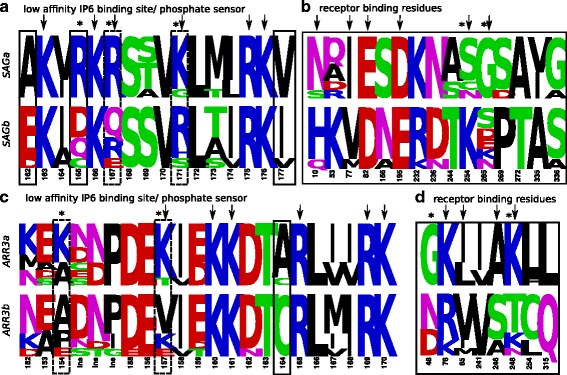
Specificity determining positions discriminating each pair of duplicated visual arrestins in teleosts. Amino acid frequency logos are shown for *SAGa* vs. *SAGb* (**a**, **b**) and for *ARR3a* vs. *ARR3b* (**c**, **d**) in teleosts. Positions that are known to directly confer a specific functionality in mammalian arrestins are marked by arrows. Of these, some mutations change the charge of the respective residue (marked with *). Positions identified by SDP analysis are highlighted by black boxes. As receptor specificity is mediated by a rather big interface, only the SDPs are shown that are known to be involved in receptor binding and their first and second order neighbors. Additional positions that show differences in both groups (manually identified) and might be associated with the respective function are highlighted with a dotted box. See [2] (pos. 10, 77, 81/76, 82, 319/313), [33] (pos. 195, 254/248), [23] (pos. 52, 54/49, 265), [17] (pos. 157, 273), [19] (pos. 90/85, 244, 267, 246/240, 261/255) for references of receptor binding residues, [19] (pos. 171/165, 175/169) for phosphate binding and [118] (pos. 163/157, 166/160, 167/161) for IP6 binding residues. The numbering refers to the position numbers in bovine *SAG* and *ARR3*, respectively. Results are also summarized in Additional file 6. The figure was created with Weblogo [116]. Ins - Insertion in comparison to reference

The paralogous pairs of *SAGa* and *SAGb* as well as *ARR3a* and *ARR3b* persisted in all investigated teleost species and evolved with similar rates since their emergence. *SAGa*s/*SAGb*s and *ARR3a*s/*ARR3b*s are recognized as separate groups in unsupervised MCA applied to alignments of *SAG* and *ARR3* in fish, respectively, emphasizing their sequence divergence. SDPs of both paralogous groups are involved in phosphate sensing and receptor binding (Fig. 7 a–d). About 17% and 13% of residues evolved under positive selection in the ancestral branches of *SAGa* and *SAGb*, respectively (Table 1, Additional file 6). See Additional file 1: Appendix 3 for a detailed description of the evolution of *SAGb* and *ARR3b* in different orders of teleosts.

### Loss or pseudogenization of *ARR3* in Afrotheria, Xenarthra, and the common shrew

**Fig. 8:**
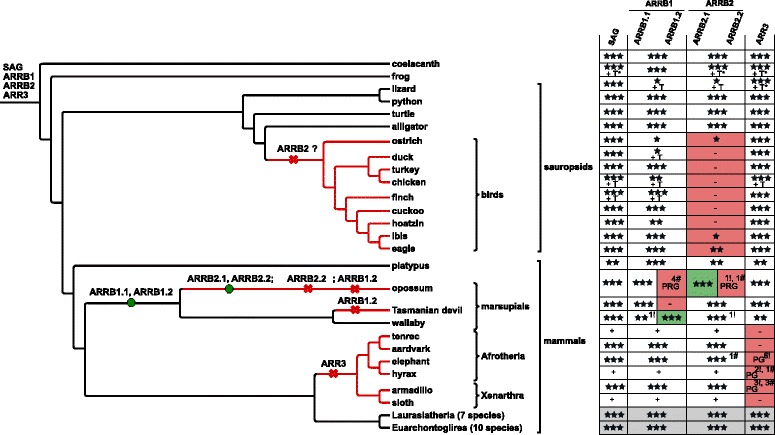
Duplication and deletion of arrestin paralogs in lobe-finned fish. *ARRB2* could not be detected in the genomes and transcriptomes of birds (see Additional file 1: Table S1 for other 41 investigated bird species). Additional omics-data was investigated for sauropsids. The gene loss/duplication pattern was simplified for the monophyletic groups highlighted in light grey (see Additional file 1: Appendix 4). See caption of Fig. 3/6 for description of symbols. The exclamation mark (!) indicates the number of stop codons contained in the exon annotation, while plus (+) indicates that gene (parts) are encoded within the respective genome, but were not annotated in detail. Note that the order of the *ARRB2.2* and *ARRB1.2* losses is arbitrary. The phylogenetic tree was created using Treegraph 2.0.54 [114]. PG - pseudogene; PRG - pseudo-retrogene

Within the second clade of bony fish, the lobe-finned fish, a single gene for each of the four paralogs is retained with a few exceptions: (1) Loss or pseudogenization of *ARR3* in Afrotheria, Xenarthra and common shrew (*Sorex araneus*); (2) Retrogene formation and pseudogenization of *ARRB1* and *ARRB2* in marsupials; (3) likely loss of *ARRB2* in birds (Fig. 8).

In the genomes of African elephant, *Loxodonta africana*, and rock hyrax, *Procavia capensis*, *ARR3* orthologs are degraded to pseudogenes to different extents (see Additional file 1: Appendix 4 for details about the investigation of the *ARR3* locus in the respective species). Both species belong to the superorder Afrotheria. In contrast, *ARR3* is completely lost in the genome of armadillo, *Dasypus novemcinctus*, which belongs to the taxonomic group of Xenarthra. An independent degradation of *ARR3* to a pseudogene was observed in the genome of common shrew (see Additional file 1: Appendix 4).

### Retrogene formation and pseudogenization of *ARRB1*/*ARRB2* in marsupials

Another peculiarity within mammals is the identification of an additional *ARRB1* and *ARRB2* gene in the marsupial genomes of opossum and wallaby (*Macropus eugenii*) (Fig. 8). Both genes are encoded by a single exon, the main characteristic of a retrogene. While the *ARRB1.2* retrogene seems functional in wallaby (Additional file 1: Figure S6), it has turned into a pseudo-retrogene in opossum indicated by four frame shift mutations within the potentially protein-coding region. Applying the parsimonious principle, we assume that a processed *ARRB1*-mRNA was inserted into the nuclear genome of the common ancestor of both species between 82-177 mya before split of Didelphimorphia and Australidelphia [37] (Fig. 8, Additional file 1: Figure S7). Remarkably, both *ARRB1.2* retrogenes share high conservation within the putative 5’ untranslated region as annotated by Ensembl for wallaby multi-exon *ARRB1.1* (Additional file 1: Figure S6). In the third investigated marsupialian genome, the Tasmanian devil (*Sarcophilus harisii*), the *ARRB1* retrogene is completely lost.

Additionally, an *ARRB2* retrogene was inserted within a cluster of zinc-finger transcription factors on chromosome 3 in the lineage leading to opossum. However, the retrogene turned into a pseudogene containing a premature stop codon and an insertion resulting in a frame-shift mutation (Fig. 8).

### Possible loss of *ARRB2* in birds

To our surprise, hardly any fragments of *ARRB2* were detected in bird genomes or lizard (*Anolis carolinensis*), while the respective ortholog was easily detectable in the genomes of other Sauropsids, e.g. alligator (*Alligator mississippiensis*), turtle (*Pelodiscus sinensis*) and python (*Python molurus*). This raised the possibility of a loss of the *ARRB2* gene within these species. Extensive homology search in 50 bird genomes retrieved only five species that harbor two or more complete exons of this 15 exon gene *ARRB2* (Additional file 6). All detected exons have a high sequence identity to orthologous exons in turtle (on average 91.3%, at least 83.9%). The potential loss was further tested by investigating genomic synteny of *ARRB2* and expression of *ARRB2* in transcriptome data (see Additional file 1: Appendix 5 for details). Neither strategy provided evidence to reject the hypothesis that *ARRB2* has been lost in birds. In contrast, a query of the NCBI EST database retrieved both non-visual arrestin transcripts in lizard confirming the integrity of the *ARRB2* gene in reptiles.

### Loss and gain of functional elements

**Fig. 9:**
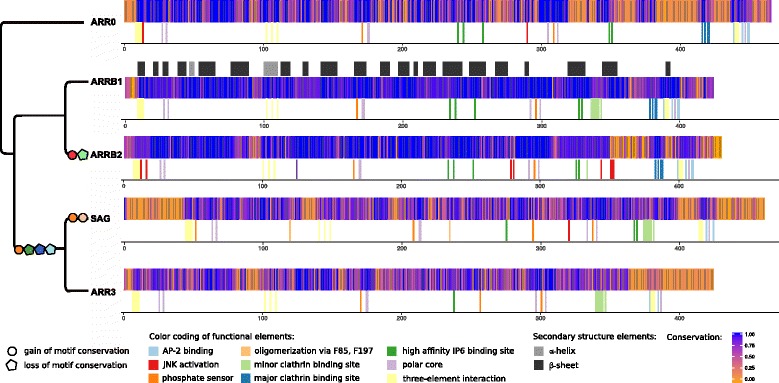
Changes in conservation patterns and functional motifs of arrestins. Conservation of alignment positions of the individual orthology groups is shown. The conservation was calculated according to the Method of Karlin [110] using AACon [111] for each orthology group separately. Sequences with a coverage < 90% as well as all lamprey sequences were excluded. Functional motifs characterized in one or several paralogs were projected onto the individual alignments solely based on sequence homology. Putative loss (*pentagon*) and gain (*circle*) events based on conservation of the respective motifs were projected onto a simplified arrestin gene tree. The order of motif gain and loss on the respective branch was chosen arbitrarily. Positions were not marked if they did not conserve the amino acid known to be part of the motif in that orthology group in a majority of representatives. Some positions are marked although their conservation is restricted to a phylogenetic group as indicated by their lower conservation score (e.g. oligomerization is specific for Sarcopterygii *SAG*). The secondary structure based on the crystal structure of 1G4R (Fig. 1) is mapped onto the alignment of *ARRB1*. Note that only a selection of known motifs are shown

Scanning the Pfam28.0 database using hmmscan confirmed that more than 95% of all annotated deuterostome arrestins possess an arrestin_C and an arrestin_N domain (see Additional file 1: Appendix 6 for details about other domains). As expected, known key functional motifs such as the phosphate sensing residues [6], the polar core residues [20], the residues involved in the three element interaction, and the sequence of the receptor-binding finger loop [32] are conserved in all *ARR0* and vertebrate arrestins (Fig. 9). The great majority of residues of all arrestins evolved under strong purifying selection and are highly conserved. However, recently duplicated paralogs can behave differently in respect to conservation and selection (Additional file 6).

We propose that the duplication of *ARR0* led to the emergence of new functionalities that are commonly conserved in the respective orthology group in vertebrates. For example, arrestin-3 binds and activates JNK3, while arrestin-2 does not [38–40]. The residues S13 and C17 previously identified to mediate JNK3 binding and activation are strictly conserved in all *ARRB2* except for lamprey and *ARRB2b* pufferfish [41] (Fig. 9). *ARR0* not only shows residues conserved among non-visual arrestins, but also paralog-specific positions with *ARRB1* and *ARRB2* in the N-terminal 25 residues. The conservation of most other positions known to mediate JNK activation is restricted to a phylogenetic group of *ARRB2* such as conservation of H350D351H352 in mammals and of L278xS280 in lobe-finned fish, respectively [40]. An exception is position V343 in the C domain of arrestin, which is conserved in all *ARRB2* except Otomorpha *ARRB2a*. Interestingly, all sea urchin *ARR0.1* sequences carry a conserved valine here, while all other *ARR0* carry threonine at the homologous position, which is characteristic for arrestin-2.

**Fig. 10:**
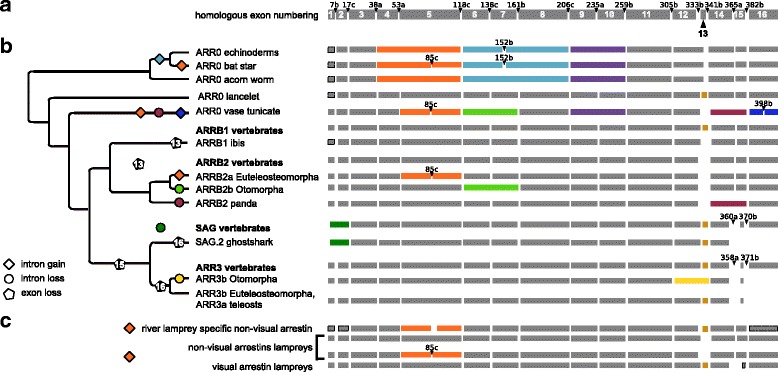
Evolutionary changes in exon-intron structure of arrestins. **a** - Exon-intron structure of the bovine *ARRB1* gene. Exon and intron numbering is imposed onto arrestin homologs by sequence alignment. Positions of introns refer to their position on the amino acid sequence of cow arrestin-2 with a-c indicating their position after the first, second or third base of the codon, respectively. **b** - Exon-intron structure of arrestins (right hand side) is associated with a simplified gene tree (left hand side). Exons are shown as grey and colorful boxes, whereby homologous regions are “aligned” below each other. Colored exons highlight differences in exon-intron structure (intron gain, intron loss, exon loss). Changes in intron positions in comparison to the reference amino acid sequence of cow arrestin-2 are given whenever deviating except for the positions surrounding exons 13 and 15, which occasionally deviated by few nucleotides in our annotation (see Additional file 1: Appendix 7). Information about the corresponding exons was not available in the genomes if boxes are surrounded by a dotted line, but are assumed to be the same as in the 1:1 ortholog of the closest relative. If an unequivocal scenario of intron loss or gain is in accordance with the maximum parsimony principle, these events are indicated in the phylogenetic tree. Paralogs of species that share the exon-intron structure are summarized to phylogenetic clades, e.g. *ARRB1* vertebrates. Structural differences in comparison to the family are shown right below associated with the corresponding species or phylogenetic clade. Losses of coding sequence (exons) are indicated by black pentagons with respective exons given as a number in the pentagon. The phylogenetic tree was created using Treegraph 2.0.54 [114]. **c** - Exon-intron structure of lamprey arrestins. Note that the length of the exon boxes is drawn to scale

In both visual arrestins, the high affinity IP6 binding site, the AP-2 binding site and the major clathrin binding site are not conserved or loosely conserved, in contrast to non-visual arrestins (Fig. 9). Other key mutations in comparison to *ARR0* involve A253D, which was hypothesized to weaken the hydrogen bond network of the pre-activated state in comparison to non-visual arrestins [42]. An additional phosphate-binding residue, R18, is conserved in all *SAG* sequences [24]. The residues F85 and F197, which are known to be involved in oligomerization of *SAG* [43] are strictly conserved in *SAG* of the lobe-finned fish. The C-terminus of teleost *ARR3* is shorter than in *ARR3* of other vertebrates. For example, the C-terminus of *ARR3a* and *ARR3b* in zebrafish is 31 and 24 amino acids, respectively, shorter than the C-terminus of *ARR3* in spotted gar. The residues missing in zebrafish are known to be responsible for the three-element interaction, AP-2 binding and contribute an arginine to the polar core [10] (Figs. 9 and 10).

Fine-tuning of receptor endocytosis is regulated by various post-translational modifications at positions conserved within but not across orthology groups (not shown). Phosphorylation of S412 of *ARRB1* regulates clathrin binding and endocytosis [44]; phosphorylation of S/T360 in *ARRB2* regulates clathrin-mediated internalization [45]; nitrolysation of C409 in *ARRB2* promotes binding to clathrin and AP-2 [46]. Other positions known to be phosphorylated and involved in the regulation of internalization, binding of clathrin (T382 in *ARRB2*) and interaction with mu2-adaptin (Y54 in *ARRB1*) are mammalian-specific and, thus, represent recent evolutionary innovations. Additional information on conservation of possible isoforms can be found in Additional file 1: Appendix 7 and Additional file 6.

### Hotspot of exon gain/loss at positions determining receptor specificity

The exon-intron structure of the vertebrate arrestin paralogs is highly conserved, preserving the majority of exon-intron boundaries of their last common ancestor, *ARR0* (Fig. 10). Nevertheless, changes in gene structure including loss of coding sequence, intron gain or loss are much more frequent in the arrestin gene family than in other vertebrate gene families [47]. In accordance with the propensity for these events in paralogous gene families as discussed by [48, 49], these gene structure changes mainly occurred within arrestin genes that underwent a tandem duplication (exemplified by loss of exon 16 in *SAG* of ghost shark) or WGDs (loss of exon 16 in *ARR3* of teleosts, gain of intron 85c in *ARRB2a* of Euteleosteomorpha, loss of intron 138c in *ARRB2b* and of intron 333b in *ARR3b* of Otomorpha). This can be further illustrated by to the emergence of the four arrestin paralogs by 2R-WGD from *ARR0* accompanied by at least one intron loss event (intron 7b) in *SAG* and a loss of coding sequence in the ancestor of *SAG* and *ARR3*, as well as in *ARRB2* (exons 15 and 13, respectively) (Fig. 10 b, Additional file 1: Figure S7). Interestingly, we observed the gain of intron 85c between 148-230 mya in the ancestor of Euteleosteomorpha, a branch of teleosts, for which frequent intron gains were described previously for several GPCRs and the serpin gene family [47, 50, 51].

A parsimony reconstruction of intron loss and gain points out a hotspot of intron gain at position 85c (Fig. 10 b/c, see Additional file 1: Appendix 8 for details). Introns were gained five times independently at position 85c of deuterostome arrestins. Four of these events occurred at the exact same position, while the exact position of intron gain in the river lamprey-specific non-visual arrestin cannot be resolved with the available data. This paralog is excluded from the following conclusions. Two intron gains occurred within vertebrates, a very rare event for this clade [47, 52].

**Fig. 11:**
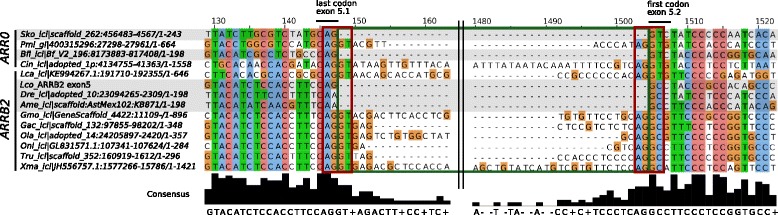
Alignment of exon-intron borders after insertion of intron 85c into exon 5. Intron 85c is found in *ARR0* of bat star (Pmi) and vase tunicate (Cin), but not in acorn worm (Sko) or lancelet (Bfl) (*highlighted in grey*). Exon 5 of one of the non-visual arrestins in lampreys (shown: Lca) as well as in *ARRB2* in all Euteleosteomorpha (Gmo, Gac, Ola, Oni, Tru, Xma) is split into two parts, denoted as 5.1 and 5.2. In contrast, exon 5 of *ARRB2* is not split in Otomorpha (Dre, Ame) and spotted gar (Lco) (*grey*). Only the 5’- and 3’-parts of the intron sequences are shown (*green box*), while the larger inner region is left out being non-informative (*black lines*). The proto-splice site motif ‘AGGY’ is conserved for all species genes shown except for Otomorpha (‘AAGC’). The alignment was visualized with Jalview 2.8.1 [93]

Introns are known to preferentially insert into sequences that carry an upstream AG and a downstream G in respect to the insertion site. This site, “AG |*intron*|GY”, has been termed protosplice site in literature [53], whereby | denotes a splice site. Alignment of the intron-containing paralogs with their intron-deficient orthologs of closely related species revealed a prevalence of intron gain at this position caused by the existence of a protosplice site in all intron-containing paralogs (Fig. 11). Newly gained intron sequences of the respective arrestin paralogs did not have any apparent sequence homology. This architecture suggests intron-insertion mediated by an endonuclease, which cuts downstream of AGGY in the exon thereby producing sticky ends. A transposon than inserts into this locus [54]. Missing nucleotides are probably filled up by a DNA polymerase resulting in two identical motifs at the 5’- and 3’-end of the inserted sequence establishing the canonical splice site AG |GT-intron-AG |GY. There is no codon that spans exons 5.1 and 5.2, the first and the second part of exon 5, respectively. The last codon of exon 5.1, CAG, is translated into glutamine, which is conserved in all but two inspected arrestins (Fig. 10). The first codon of exon 5.2 is much less conserved translating into different non-polar, aliphatic amino acids (L, M, I, A, V) in visual arrestins (V90 in *SAG*, V85 in *ARR3*) and into small amino-acids (A, S) in non-visual arrestins with three exceptions (S86 in *ARRB1*, A87 in *ARRB2*).

Interestingly, V90 in bovine arrestin-1 is not surface-exposed. It is located between the two *β*-sheets of the N domain, making contacts with several other hydrophobic residues [55]. Its substitution with a small side chain residue characteristic for non-visual arrestins (A or S) enables arrestin-1 binding to non-cognate M2 muscarinic receptor [55]. Therefore, large hydrophobic residue in this position likely makes the N domain more rigid, predisposing an arrestin to be more GPCR subtype-specific [19, 56].

## Discussion

We uncovered the complex duplication and deletion history of the arrestin family in deuterostomes based on a careful evaluation of genomic information. Our approach enhanced by manual curation outperforms conventional strategies that rely on uncurated databases to infer orthology relationships (OrthoDB).

We show that the paralog counts differ for 25 species (44%) between the updated annotation and OrthoDB, a frequently used database that is considered to be fairly complete (Fig. 2). The majority of deviations is caused by an underestimation of paralog counts in OrthoDB exemplifying the incompleteness of this database. Although patterns of absence and presence of paralogs are conveniently presented in OrthoDB, it remains an open problem to distinguish paralog losses from missing information for biological and technical reasons in automated procedures. Biological reasons are high degree of divergence, duplications, and pseudogenizations. Over- and especially underprediction of paralogs is mostly caused by technical issues, e.g. due to sequencing techniques, sequencing coverage, annotation and assembly strategies. Particular care should be taken when inferring the expected number of paralogs from the maximum number of paralogs in the database, proposing an exaggerated rate of losses. A successful strategy to perform gene family annotation uses a whole genome homology search, a priori information about genome duplications and exon-intron structure. Consideration of lowly sampled taxonomic groups phylogenetically close to gene loss and gain events is critical to resolve the genealogy. Information extracted from current protein databases can thus just deliver a preliminary overview on paralog counts and orthology relationships. Here, we establish a high-quality standard of a small curated data set that can be used as a benchmark for annotation and orthology prediction tools. As homology search methods propagate erroneous annotations, the improvement of existing annotations and methodology for annotation and orthology predictions is a necessity in computational biology [57, 58].

The majority of deuterostome arrestin paralogs arose by WGD, either at the root of vertebrates or at the root of teleosts. These events promoted the acquisition of new functions and changes in exon-intron structure of arrestins.

The 2R-WGD led to the emergence of the four arrestin paralogs from a prototypical arrestin presumably similar to *ARR0* in vase tunicate in accordance to [30, 59]. Arrestins are in line with several other gene families of the phototransduction cascade, e.g. opsins, G protein-coupled receptor kinases and transducins, all of which expanded by the basal vertebrate tetraploidizations and thus paved the way for the development of a sophisticated visual system in the vertebrate clade [59]. Some studies place the 2R-WGD after the split of jawless fish and jawed vertebrates suggesting independent duplications in the lamprey-lineage [60, 61], other studies argue that both 2R-WGDs took place at the root of jawed vertebrates, followed by an immediate split of both groups [62, 63]. The current study of one gene family, also cannot resolve this controversy. It remains unclear, therefore, whether the lamprey arrestins represent 1:1 orthologs to the vertebrate arrestins or arose from independent duplications after a shared first WGD.

A third WGD resulted in further increase in the number of arrestin paralogs in teleosts. Visual arrestins were more readily retained after duplication than non-visual arrestins. [3] made the first attempt to functionally characterize *ARR3a* and *ARR3b* in zebrafish, which they found specifically expressed in the outer layer of either M- and L-cones (*ARR3a*) or of S- and UV-sensitive cones (*ARR3b*).

In addition to spatial subfunctionalization, we propose that expansion and diversification of opsins is paralleled by functional diversification of *ARR3a* and *ARR3b*. This is supported by [3], and our comparative analysis revealing mutations of receptor binding residues. As a second example of functional subfunctionalization, we find SDPs of phosphate and IP6 binding residues affecting binding specificity, in agreement with functional studies showing that *SAGa* and *SAGb* have different binding affinities for phosphorylated rhodopsin in carp [64].

The duplicated non-visual arrestins, *ARRB2a* and *ARRB2b*, were shown to have similar functions in modulating the distribution of the chemokine ligand Cxcl12a, but different spatial expression patterns in zebrafish primordial germ cells [65]. These paralogs are nearly identical in zebrafish. In contrast, *ARRB2* of stickleback and pufferfish carry mutations in key functional motifs presumably impairing their function.

In addition to 3R-WGDs, local duplications such as tandem duplications or retrogene insertions contributed to the emergence of more arrestin paralogs within smaller classes or infraclasses. The sea urchin-specific tandem duplication of *ARR0* seems to be in line with the over-representation of arrestin-interaction partners such as the secretin-like GPCR superfamily [66] and the rhodopsin-type GPCRs expressed in sensory appendages and the nervous system in purple sea urchin [67]. Furthermore, the Ras-superfamiliy of G proteins regulated by arrestins, as well as receptor protein tyrosine phosphatases regulating arrestin binding to GPCRs, are expanded in sea urchin hinting at a general expansion of molecules involved in GPCR signaling [68, 69]. *ARR0.1* is suggested to have acquired a new function in connection with receptor binding, enhanced phosphate sensing and, possibly, reduced binding to the clathrin adapter protein AP-2.

In conclusion, a common theme for fine tuning or specialization of arrestins after duplication seem to be the following two degrees of freedom: receptor binding and phosphate sensing.

In addition to expansions of the arrestin system we also found some losses, in particular, the well-documented pseudogenization/loss of *ARR3* in Afrotheria, Xenarthra, and common shrew. Less obvious is the possible loss of *ARRB2* in birds. The study, simply based on comparative genomics, is strongly dependent on genome coverage and the quality of the available assemblies. As these differ widely among vertebrate genomes, we were particularly cautious hypothesizing about exon or gene losses. Whenever possible, multiple data sources and strategies were used. When available, we took into account additional transcriptome data and investigated arrestin genes in additional, closely related species to obtain information on synteny. Nevertheless, we cannot rule out that individual genes, such as bird *ARRB2* escaped sequencing or assembly even after having considered 50 bird genomes in this study. The incompleteness of avian genomes, and the difficulty of sequencing certain regions in these genomes is a well known, albeit poorly understood, phenomenon [70–72]. Regions known to cause difficulties in sequencing and assembly are heterochromatin and repeat regions [73], and also bird microchromosomes in general. It remains unclear, therefore, whether the single, detected exons of *ARRB2* in birds and of *ARR3* in hedgehog are remnants of a pseudogene or of an intact gene. The exons we identified for *ARRB2* have a high sequence conservation in comparison to mammalian *ARRB2*, cover different parts of the gene and are generally situated on short contigs suggesting difficulties in sequencing and/or assembly. On the other hand, we did not detect any transcripts of *ARRB2* in various transcriptome data sets representing different tissues and developmental states of several bird species. One has to bear in mind, though, that the expression of the ubiquitous arrestin-3 could be too low to detect its transcripts as arrestin-3’s expression is 10-20-times lower than that of arrestin-2 in most mammalian cells [74].

In contrast, the loss of *ARR3* could be shown explicitly for Afrotheria and Xenarthra based on the synteny information. *ARR3* is specifically expressed in cones and pinealocytes [75], where it inactivates phosphorylated cone opsin and interacts with additional binding partners e.g. Mdm2, JNK3 [76], Als2Cr4 [77] or MKK4, ASK1 [4] acting as a scaffolding molecule. Whereas it is the only visual arrestin expressed in L- and M-cones in humans and several primates [78], both, *SAG* and *ARR3*, are expressed in primate S-cones [78] and all mouse cones [22].

The evolutionary need for *ARR3* has already been discussed in the literature emphasizing differences between *SAG* and *ARR3*, namely the ability of *SAG* to self-assemble and the transient binding affinity of *ARR3* to receptors [4]. *ARR3* null mice have an impaired contrast sensitivity and visual acuity when young, while their cones seem to degenerate slower with increasing age in comparison to wild type (WT) mice as shown recently [79]. However, other studies in mice suggest that the arrestin-4 function can be fulfilled by arrestin-1. The response of S-dominant cones of *ARR3* null mice to light stimuli is similar to WT mice, while recovery from flashes is greatly slowed down in *SAG*/*ARR3* double-knock out mice [22]. The authors concluded that at least one visual arrestin is necessary for inactivation of S- or M-opsin in mice. Additionally, [80] showed that arrestin-1 can inactivate S-opsin metaII in transgenic mice expressing S-opsin instead of rhodopsin in rods, although arrestin-1 does not seem to be necessary for dim-flash response in WT cones. Thus, these studies suggest that arrestin-1 could take over the arrestin-4 function if expressed in cones, which is consistent with the loss of *ARR3* reported in our study. Alternatively, other adaptions could have evolved in Afrotheria and Xenarthra to compensate for the loss of *ARR3*.

Although the evolution of e.g. mammalian arrestins has been examined previously [9], the present study uncovered numerous previously unreported gene gain and loss events within arrestins in deuterostomes. Identification of residues that determine specificity and are positively selected after duplication was made possible by a high quality alignment obtained by genome inquiries, dense species sampling and consideration of fragmented loci from poorly assembled genomes. The residues identified during this study as evolutionary “adjusting screws” are candidate positions for construction of biased arrestins that were already approved by nature.

## Methods

### Database scans

For performing the homology search, we generated a pHMM using jackhmmer with an alignment of the four human arrestins as input querying the UniProtKB database (accessed via https://www.ebi.ac.uk/Tools/hmmer/, 30 June 2017). The level of homology retrieved with different jackhmmer iterations was checked by comparing to the results of a homology search with the arrestin_N domain and arrestin_C domain HMM as downloaded from Pfam 31.0 (PF00339, PF02752, E-value < 0) in order to obtain a good overlap of both strategies. The full-length set of homologs obtained from UniProtKB was filtered according to length (422 > length > 195, *μ* +- *σ*), E-value (< 0) and identity of the full-length sequences for each species separately (< 80%). The identity filter cut-off was chosen to balance the removal of isoforms and retention of paralogs. We obtained a set of 2962 sequences, of which 2348 contained at least one arrestin_N and one arrestin_C domain (Additional file 1: Figure S5). 142 sequences did not have either of both domains and were excluded. We proceeded with the full-length sequences of this set under exclusion of hits that were not assigned to one specific species, for phylogenetic inference, and for reporting paralog counts projected on the NCBI phylogeny. In order to exclude effects on phylogenetic inference that can arise from aligning sequences that are not homologous in full-length, we additionally generated individual domain sets for the arrestin_N and arrestin_C domain, respectively, and also proceeded to phylogenetic inference. These sets consist of the respective Pfam model hit in the UniProtKB database restricted to the actual hmmsearch hit length. Both sets were filtered according to identity (see above).

Furthermore, we queried OrthoDB (as of Feb. 2017) with full-length arrestin pHMMs (E-value < 0) obtained with jackhmmer. OrthoDB is considered to be a high quality orthology database, which contains unique orthology group assignments for proteins of interest on a given taxonomic level. We restricted our analysis to the OrthoDB groups that are annotated on Metazoa level and retrieved 3487 hits that belong to 109 orthology groups. For better visibility while plotting, we only distinguish between groups that have more than 29 members. These 9 groups cover 88% of all sequences. Other orthology groups are denoted as “Other” (Additional file 1: Figure S2). The NCBI species tree was retrieved with the ete toolkit [81].

### Detailed gene annotation

Automated methods frequently fail to correctly predict multi-exon genes. We therefore used exon- and paralog-specific pHMMs to update the annotation of arrestin genes in different genomes of interest ([82], see next paragraph). Exon models were built from an initial, manually curated protein alignment of mammalian arrestins (see Additional file 1: Appendix 9 for details about reconstruction of the initial alignment). It was then extended by adding the translated exon sequences from arrestins successively annotated in other lobe-finned and ray-finned fishes using HMMER 3.1b1 [83]. These exon- and paralog-specific models were then handed over to the EMS pipeline [82]. Simplified, the EMS performs a two step procedure: (1) a homology search with the provided pHMMs, (2) assignment of exon- and paralog-specific hits to one paralog based on an integer linear programming formulation of the paralog-to-contig assignment problem. In contrast to other methods, the EMS pipeline considers paralogs that are fragmented over several, often short contigs and assembles these paralog fragments to a more complete annotation. The assignment of paralogs to contigs is explicitly solved and provides the starting point for manual curation.

In case of a systematic failure to detect a specific arrestin exon within a monophyletic family with the EMS pipeline, the candidate region was re-investigated with different homology-based methods. These included local blastall 2.2.26 querying a region between two exon hits with the nucleotide sequence of the missing exon(s) applying blastn or, with the protein sequence of the conceptually translated exon, respectively, applying tblastn [84]. To detect exons that differed substantially among homologous groups, we aligned the corresponding regions of at least three close relatives of one group with tba.v12 [85] and applied RNAcode 0.3 to detect conserved regions with protein coding potential [86].

### Genome versions used for the current study

Unmasked genomes were extracted from Ensembl, EnsemblPre! or Ensembl Metazoa if available and from the listed sources otherwise (Additional file 1: Table S1). For ghost shark, only a soft-masked version of the genome was available. To clarify the potential loss of *ARRB2* in birds, all available 48 bird genomes from the Avian Phylogenomics Project [87], the genomes of kiwi (*Apteryx australis mantelli*) and gold eagle (*Aquila chrysaetos*) were investigated additionally. All four arrestin paralogs were annotated in nine birds in total (ostrich, chicken, turkey, duck, finch, ibis, hoatzin, cuckoo, bald eagle). Insertions and stop codons were occasionally observed within exons of arrestin genes in genomes with low coverage and/or poor quality assemblies. We interpreted these as sequencing or assembly errors because the remainder of the protein-coding sequence was usually highly conserved, except in cases which we explicitly identified as pseudogenes in the current study (e.g. elephant *ARR3*).

### Investigation of transcriptome, EST and SRA data

Transcriptome data sets, in particular the NCBI Expressed Sequence Tag (EST) and NCBI Transcriptome Shotgun Assembly data sets, were additionally queried whenever the analysis of the corresponding genome was not conclusive. We used the NCBI webinterface to tblastn with protein sequences of closely related species as queries in these cases (Additional file 1: Table S2). Clades that were queried are “Sauropsida”, “Aves”, “Marsupilia”, “Chondrichthyes” and “Cyclostomata” [88]. NCBI Short Read Archive (SRA) was queried with the known arrestin kiwi exons against SRA data of ostrich (*Struthio camelus*) and tinamu (*Tinamus guttatus*) as well as with arrestin exons from bald eagle (*Haliaeetus leucocephalus*) against SRA data of white-tailed eagle (*Haliaeetus albicilla*) and gold eagle. As the NCBI blast did not provide a blast-database for EST data of lizard, this was built locally and queried.

### Alignment and building of phylogenetic trees

For generating a bootstrapped phylogenetic tree of the arrestin fold family, we aligned all hits obtained after filtering from the OrthoDB with clustalo 1.2.4. Next, we built an approximate maximum likelihood tree with FastTree with the -pseudo option for fragmented/gapped sequences and the following options to increase its accuracy/tree exploration -spr 4 -mlacc 2 -slownni.

For the tree of arrestins, we considered Genbank annotations of arrestins with experimental evidence (NP-entries) whenever available and more complete than the genomic annotations. The same is true for transcript evidence of arrestin paralogs. Coding DNA sequences were aligned according to codons with MACSE 1.01b [89] and further edited in mega 4.0.2 [90]. Maximum likelihood trees were built from protein sequences using PhyML 3.0.1 [91] after testing for optimal model parameters with ProtTest 3.4 allowing for the following substitution models: JTT, Dayhoff, WAG, LG, DCMut, Blosum62, an estimation of amino acid frequencies (-F), the fraction of invariable sites and a gamma-distribution (-all-distributions) [92]. Unknown amino acids were substituted by “?” in the alignment for tree building. The tree that obtained the best information content (BIC and AIC) applying ProtTest was used as starting tree for PhyML. The tree topology was validated by bootstrapping (1000 iterations unless stated otherwise). Manual inspection of the alignment revealed conservation or disruption of functional motifs previously investigated experimentally in mammals and known from literature, that were marked within the Jalview 2.8.2 alignment program ([93], Additional file 7).

Bayesian trees were constructed based on the amino acid alignment with the BEAST2 software [94] under the Birth-Death model with a relaxed molecular clock. We compared different model settings pairwise employing PathSampling [95, 96] to estimate the marginal likelihoods and calculating the Bayes factor (BF). A model was accepted if BF >3 [97]. Otherwise, the simpler model was chosen. The model settings differed in their birth-death priors and regarding estimation or fixation of different priors to specific values, while using the parameters determined with ProtTest as site model parameters.

Here, the best model had the following parameters: Relaxed Clock Log normal, birthRate Uniform, relative Death rate *β*-distributed (*alpha* = 1, *beta* = 10). For every model setting, several chains were combined after confirming that they converged to the same set of parameters with the help of Tracer v1.6 [98] and logcombiner. Trees were analyzed with treeannotator and visualized in FigTree [99].

### Identification of SDPs

For identification of SDPs of closely related paralogs that arose from a recent duplication, respective sequences were grouped, aligned and filtered to contain a redundancy < 98% and coverage > 70%. The following groups were investigated: teleost *SAGa, b*, teleost *ARR3a, b*, teleost *ARRB2a, b*, all *ARR0* including sea urchin *ARR0.1*. The filtered alignments were analyzed with four complementary SDP detection tools, the entropy-based sequence harmony approach (SH) [100, 101], the machine-learning approach multi-RELIEF [102, 103], Xdet, which is based on analysis of mutational behavior [104] and S3det based on MCA [105]. The first two approaches were run via the webserver [106], while the latter two are implemented in the program jdet 1.4.5. Positions retrieved with the default values of the respective programs (exception: S3det -m 2) were filtered according to the following, conservative cut-offs: SH z-scores < -6, multi-RELIEF-scores > 0.7 and Xdet-scores < 0.6. Group distinction was computed automatically (unsupervised) in S3det except for teleost *ARRB2*. Positions were only considered as specificity determining if they were retrieved with at least two of the four methods (see Additional file 6 for detailed results).

### Testing for natural selection

To test for natural selection, we constructed alignments of coding DNA sequence restricted to specific sub-branches of interest. Regions encoding frame shift mutations, containing stop codons or gaps were excluded from further analysis. We excluded potential recombinant sequences by testing for recombination in the group alignments with the RDP4 software [107] (*SAGa, b* zebrafish, *ARR3* stickleback). We assume that recombination and gene conversion can only occur within the same species and thus excluded incomplete lineage sorting for the species considered. Positive selection was tested on predefined foreground branches with the branch-site model of codeml inside the PAML program [108] (kappa to be estimated, F3X4 and Codon table tested as Codon frequency models). The significance of difference of the maximum log-likelihoods of the null model (*w*2=1) and the alternative model (*w*2≥1) was assessed by comparing the results of the likelihood ratio test with the $\tilde \chi ^{2}$ distribution of p-values (<0.05). In case that the alternative model was significantly better than the null model, specific sites under positive selection were assessed according to the significance levels of the BEB method. Additionally, we performed bootstrapping and assessed the distribution and confidence intervals of the bootstrapped estimates with the codeml_sba [109] method. Some data sets show a slightly bimodal distribution of *w*2 and/or *p*1 and thus obtained rather uncertain parameter estimates (reported as *μ*, *σ* and upper and lower quartiles in Additional file 6). The fraction of sites under positive selection (p2) was calculated as follows: *p*2=1−(*p*0+*p*1).

### Calculation of sequence conservation

Sequence conservation was calculated with the Karlin score [110] implemented in AACon [111] for alignments of individual orthology groups (*SAG*, *ARRB1*, *ARRB2*, *ARR3*) excluding lamprey sequences. To minimize the effect of missing data on conservation calculations, we filtered the alignments to contain sequences with a coverage > 90%.

The exon and intron numbering used throughout the manuscript is based on homology (refer to Fig. 10 a as reference). Positions of amino acids within the protein always refer to the homologous position in cow (*Bos taurus*, for *ARR0*, bovine *ARRB2* is considered). Mutations are also reported in this coordinate system. D297Y therefore means that tyrosine (Y) is found in the species of interest at the amino acid position homologous to position 297 of the corresponding bovine arrestin, which is aspartic acid (D). Gene names are used according to recommendations of the HUGO Gene Nomenclature Consortium.

## Additional files

Additional file 1 This file includes **Figures S1–S7**, **Table S1** (Genomes used) and **Table S2** (Transcriptome/EST data used) as well as the following Appendices: Appendix 1 — Arrestin inventories in lampreys. Appendix 2 — Annotation of arrestins in cartilaginous fish. Appendix 3 — Evolution of visual arrestins in different orders of teleosts. Appendix 4 — Investigation of the *ARR3* locus in Afrotheria, Xenarthra and common shrew. Appendix 5 — Investigation of loss of *ARRB2* in Sauropsids. Appendix 6 — Domains of deuterostome arrestins. Appendix 7 — Isoforms and changes of the conserved exon-intron structure. Appendix 8 — Parsimonious reconstruction of intron gain/loss events. Appendix 9 — Annotation of arrestins in mammals. (PDF 2380 kb)

Additional file 2 Approximate ML tree of the arrestin fold family as extracted from UniProtKB (depicted in Additional file 1: Figure S1). Hits were assigned to the arrestin fold family if they contained at least one arrestin_N or arrestin_C domain (see Methods). The tree was generated with the FastTree software and bootstrapping was performed 1000 times with SeqBoot [119]. The tree can be visualized with a tree viewer, e.g. Dendroscope [115]. (TREE 78.3 kb)

Additional file 3 ML tree of arrestins (depicted in Fig. 4). Starting information for tree reconstruction was an alignment of arrestins investigated in this study, for which sequence information was close to complete. Sequences derived from genomic annotations were substituted by sequences with experimental evidence available if these were more complete. The tree was constructed using PhyML from the amino acid sequences of arrestin paralogs from an alignment of nucleotide sequences aligned with MACSE (model JTT+G+I with *α* 1.04, p-invariable 0.05, 200x bootstrapping). The tree can be visualized with a tree viewer, e.g. Dendroscope [115]. (NW 1340 kb)

Additional file 4 Maximum clade credibility tree of arrestins. Starting from the same alignment as used for Additional file 3, we constructed a phylogenetic tree with BEAST2 [94] under the Birth Death model with a relaxed molecular clock (log normal) using the gamma site model (JTT+G+I with *α* 1.04, p-invariable 0.05). The tree can be visualized with a tree viewer, e.g. Figtree [99]. (TREE 253 kb)

Additional file 5 ML tree of arrestins excluding columns known to confer receptor binding. The tree was constructed from the same alignment as Additional file 3 deleting the columns known to confer receptor specificity according to [2, 17, 19, 23, 31–33] (model JTT+G+I with *α* 1.05, p-invariable 0.05, 200x bootstrapping). The tree can be visualized with a tree viewer, e.g. Dendroscope [115]. (NW 7.42 kb)

Additional file 6 Chromosomal locations: Chromosomal locations of arrestin genes. Table of chromosomal locations of arrestin genes in species with genomes assembled on chromosome-level. The columns “*SAG(a)*” contain the location of *SAG* for non-duplicated species (*SAGb* does not exist) or of *SAGa* for teleosts. This applies to all columns. Note that *ARR3* is located on the sex chromosome X in all mammals.
SDP *ARR0*: SDPs distinguishing *ARR0.1* from sea urchin *ARR0.2* and other *ARR0*. The classification is based on unsupervised multi-correspondence analysis of all *ARR0* with S3det. SDPs are listed that were predicted by at least two out of the four following methods: Xdet, S3det, SH and multi-RELIEF. Additionally, the functional annotations of homologous positions in bovine *ARRB1* are listed. SDP *SAG*: SDPs distinguishing *SAGa* and *SAGb* in teleosts. The classification is based on unsupervised MCA of all filtered teleost *SAG* with S3det. During filtering, *SAGb* from pufferfish and stickleback were excluded due to sequence coverage < 70%. SDPs are listed that were predicted by at least two out of the four following methods: Xdet, S3det, SH and multi-RELIEF. Additionally, the functional annotations of homologous positions in bovine *SAG* are listed.
SDP *ARRB2*: SDPs distinguishing *ARRB2a* and *ARRB2b* in teleosts. The classification was given in supervised MCA of all filtered teleost *ARRB2*. Due to redundancy > 98%, *ARRB2a* of platyfish (*Xiphophorus maculatus*), pufferfish and medaka were excluded from the analysis. SDPs are listed that were predicted by at least two out of the four following methods: Xdet, S3det, SH and multi-RELIEF. Additionally, the functional annotations of homologous positions in bovine *ARRB2* are listed.
SDP *ARR3*: SDPs distinguishing *ARR3a* and *ARR3b* in teleosts. The classification is based on unsupervised MCA of all teleost *ARR3* with S3det. SDPs are listed that were predicted by at least two out of the four following methods: Xdet, S3det, SH and multi-RELIEF. Additionally, the functional annotations of homologous positions in bovine *ARR3* are listed.
Codeml: Analysis of positive selection after arrestin duplication. Specific branches within the arrestin gene tree were tested for positive selection using the branch-site model of codeml, part of the PAML program. The null-hypothesis assumes purifying or neutral selection on the foreground and background branches, while the alternative model allows for positive selection on the foreground branch. Fractions of sites are given, that are predicted to belong to the respective classes (p) together with their dN/dS ratios (w). If the null hypothesis was rejected, sites that were under positive selection under BEB were mapped onto the respective bovine ortholog. (XLSX 44.5 kb)

Additional file 7 Alignment of deuterostome arrestins with functional annotation known from experimental studies of arrestins in mammals. Note that the naming differs from the naming used throughout the manuscript with *ARR1*, *ARR2*, *ARR3* and *ARR4* used instead of *SAG*, *ARRB1*, *ARRB2* and *ARR3*, respectively. Please load alignment with annotation file (provided at http://dx.doi.org/10.5281/zenodo.820866) in Jalview alignment viewer. (FA 44.5 kb)

## Abbreviations

BF: Bayes factor
EMS: ExonMatchSolver
EST: Expressed sequence tag
GPCR: G-protein coupled receptor
IP6: Inositol-6-phosphate
MCA: Multiple correspondence analysis
ML: Maximum likelihood
mya: Million years ago
pHMM: Profile Hidden Markov Model
SDP: Specificity determining position
SH: Sequence harmony
SRA: Short read archive
WGD: Whole genome duplication
WT: Wild type
