# Supplementary material for: Uncovering missing pieces: duplication and deletion history of arrestins in deuterostomes
Source: BMC Evol Biol. 2017 Jul 6;17:163. doi: 10.1186/s12862-017-1001-4 (PMC5501109; doi:10.1186/s12862-017-1001-4)
Supplement: Supplementary file 1 — This file includes Figures S1–S7, Table S1 (Genomes used) and Table S2 (Transcriptome/EST data used) as well as the following Appendices: Appendix 1 — Arrestin inventories in lampreys. Appendix 2 — Annotation of arrestins in cartilaginous fish. Appendix 3 — Evolution of visual arrestins in different orders of teleosts. Appendix 4 — Investigation of the ARR3 locus in Afrotheria, Xenarthra and common shrew. Appendix 5 — Investigation of loss of ARRB2 in Sauropsids. Appendix 6 — Domains of deuterostome arrestins. Appendix 7 — Isoforms and changes of the conserved exon-intron structure. Appendix 8 — Parsimonious reconstruction of intron gain/loss events. Appendix 9 — Annotation of arrestins in mammals. (PDF 2380 kb) [file 12862_2017_1001_MOESM1_ESM.pdf]

Additional file 1

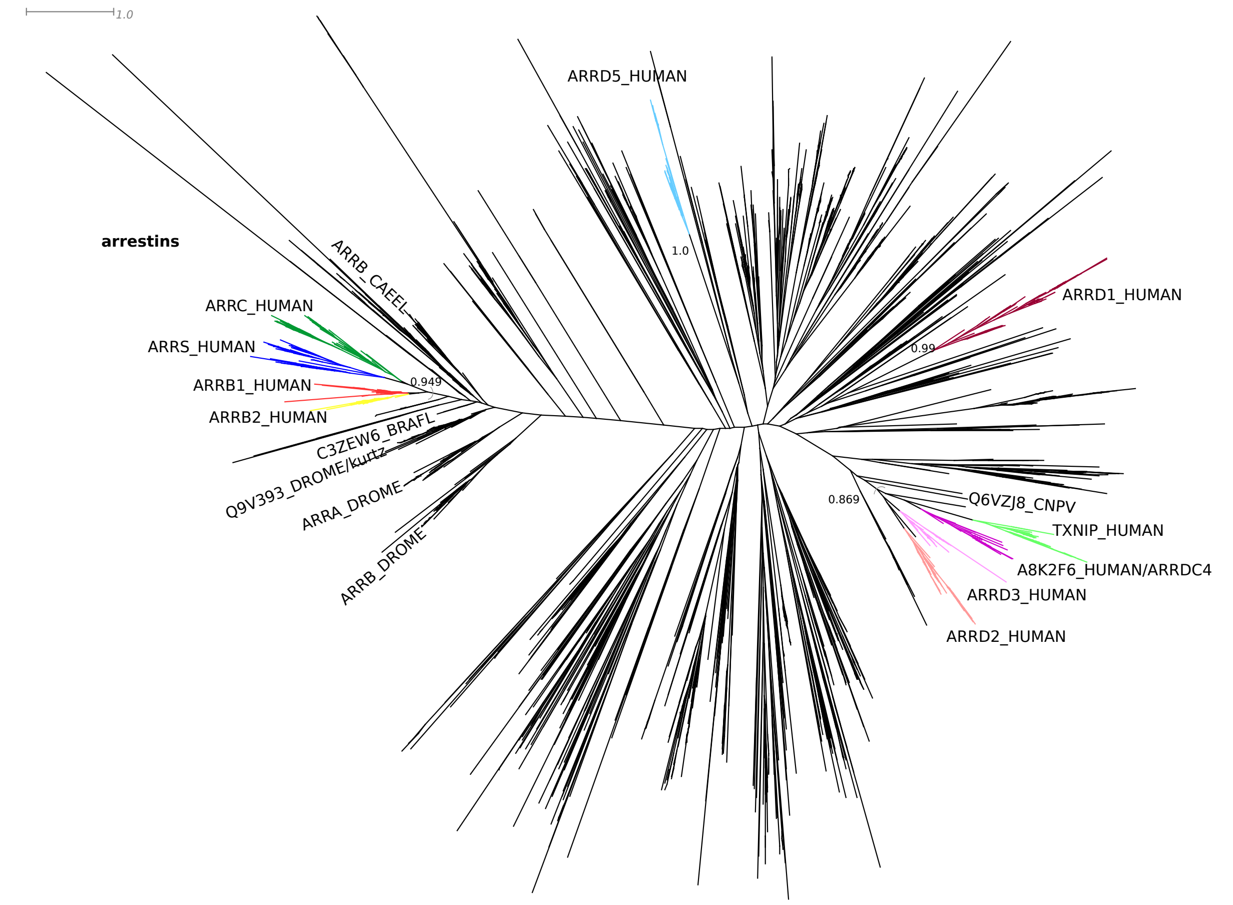

Figure S1 Approximate maximum likelihood (ML) tree of the full-length arrestin fold family members as extracted from `UniProtKB`. Hits were assigned to the arrestin fold family if they contained at least one arrestin_N or arrestin_C domain (see Methods). The tree was generated with the `FastTree` software and bootstrapping was performed 1000 times with `SeqBoot` [1]. Bootstrap values are shown for the most important splits, namely those that contain a human homolog and their vertebrate 1:1 orthologs (marked in color). Vertebrate arrestins clearly form a monophyletic group within the arrestin fold family. Some members of the arrestin fold family are labeled with their `UniProtKB` IDs. See Additional file 7 for a file with the respective tree in newick format. BRAFL - *Branchiostoma floridae*; CAEEL - *Caenorhabditis elegans*; CNPV - *Canarypox virus*; DROME - *Drosophila melanogaster*.

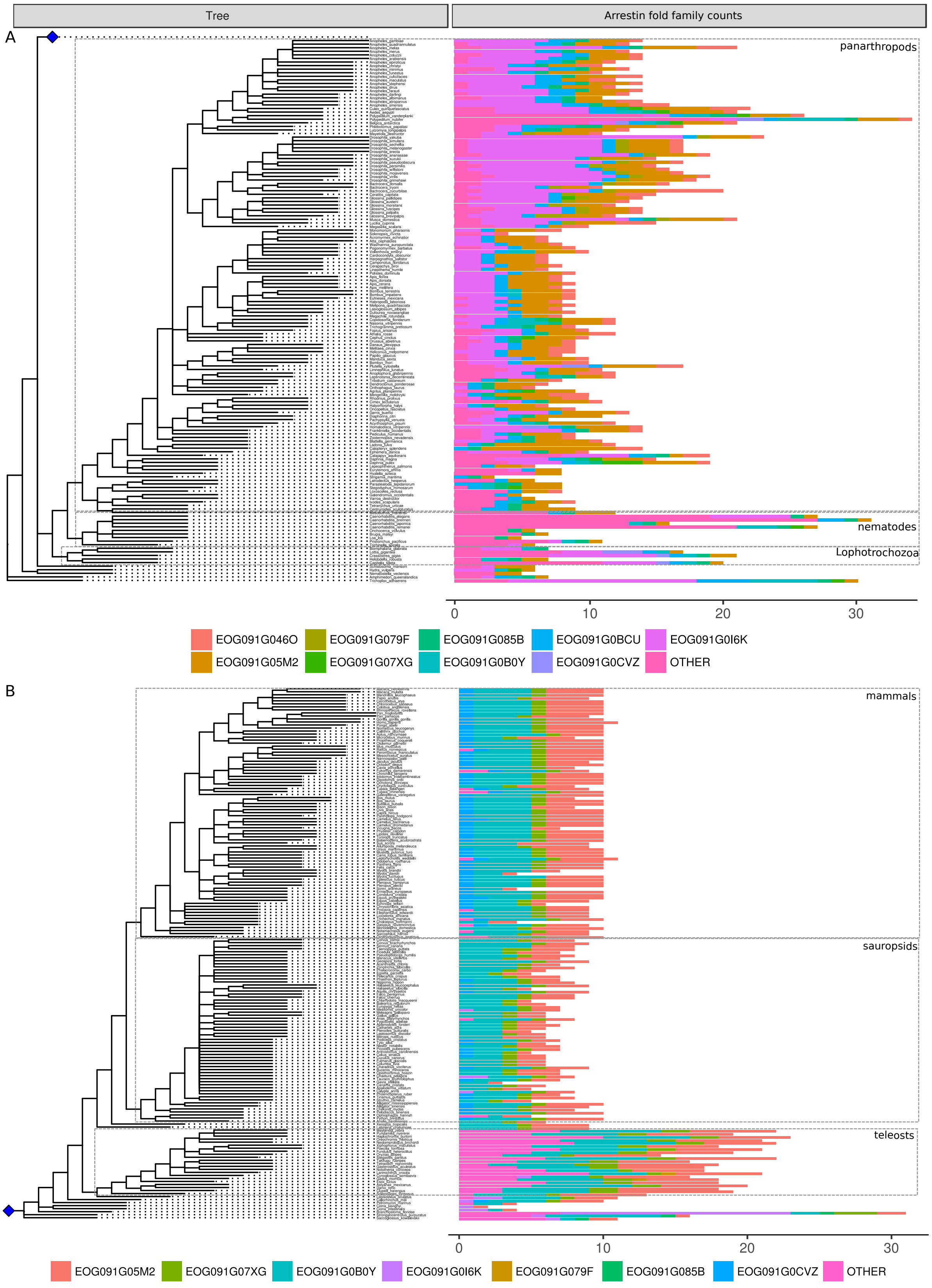

Figure S2 Abundance of arrestin fold family members in Metazoa and orthology assignment according to `OrthoDB`. Hits were mapped to the NCBI taxonomy of Metazoa. Deuterostomes are represented by a blue diamond in A and extensively shown in B. The color coding corresponds to different orthology groups. Note that groups with $<$ 29 members were collapsed to the single group "Other".

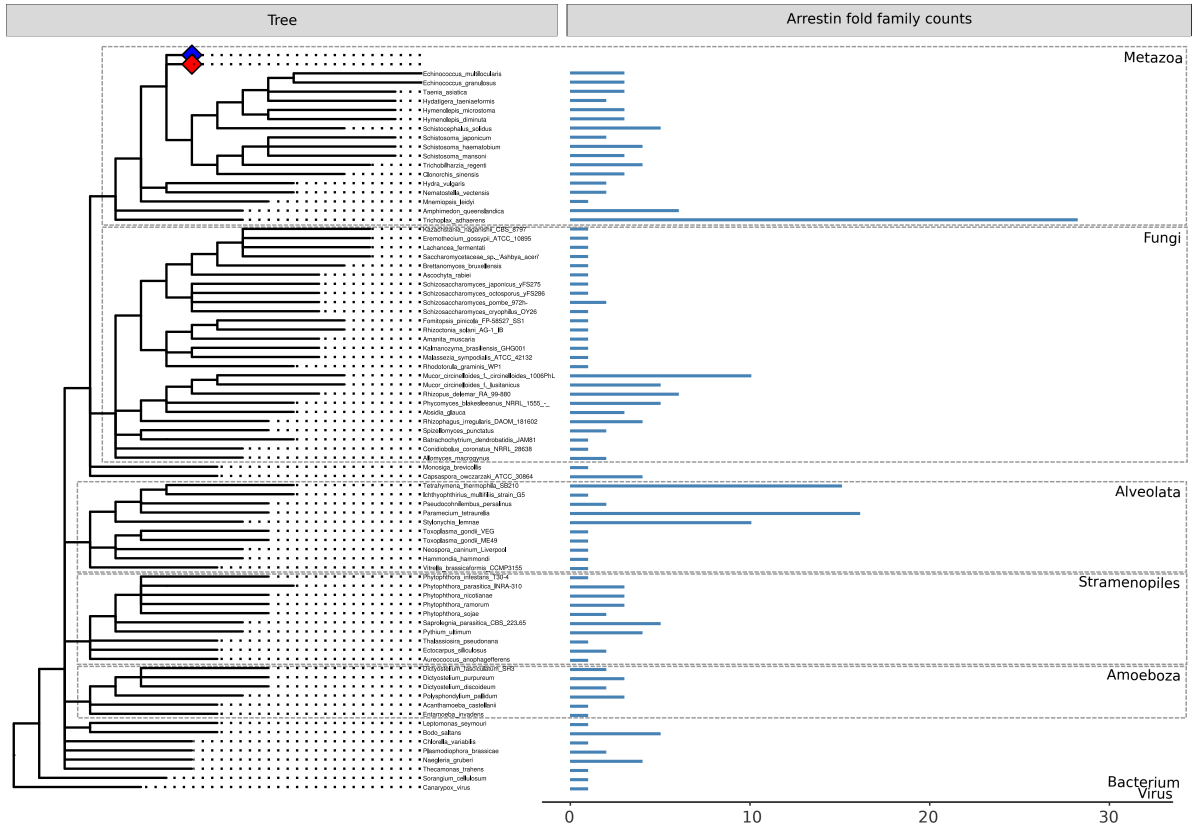

Figure S3 Abundance of arrestin fold family members in different domains of life according to `UniProtKB`**.** Hits were assigned to the arrestin fold family if they contained at least one arrestin_N or arrestin_C domain (see Methods) and were mapped to the NCBI taxonomy. The blue and red diamond simplify the groups of protostomes and deuterostomes, respectively (see Fig. 4 for details).

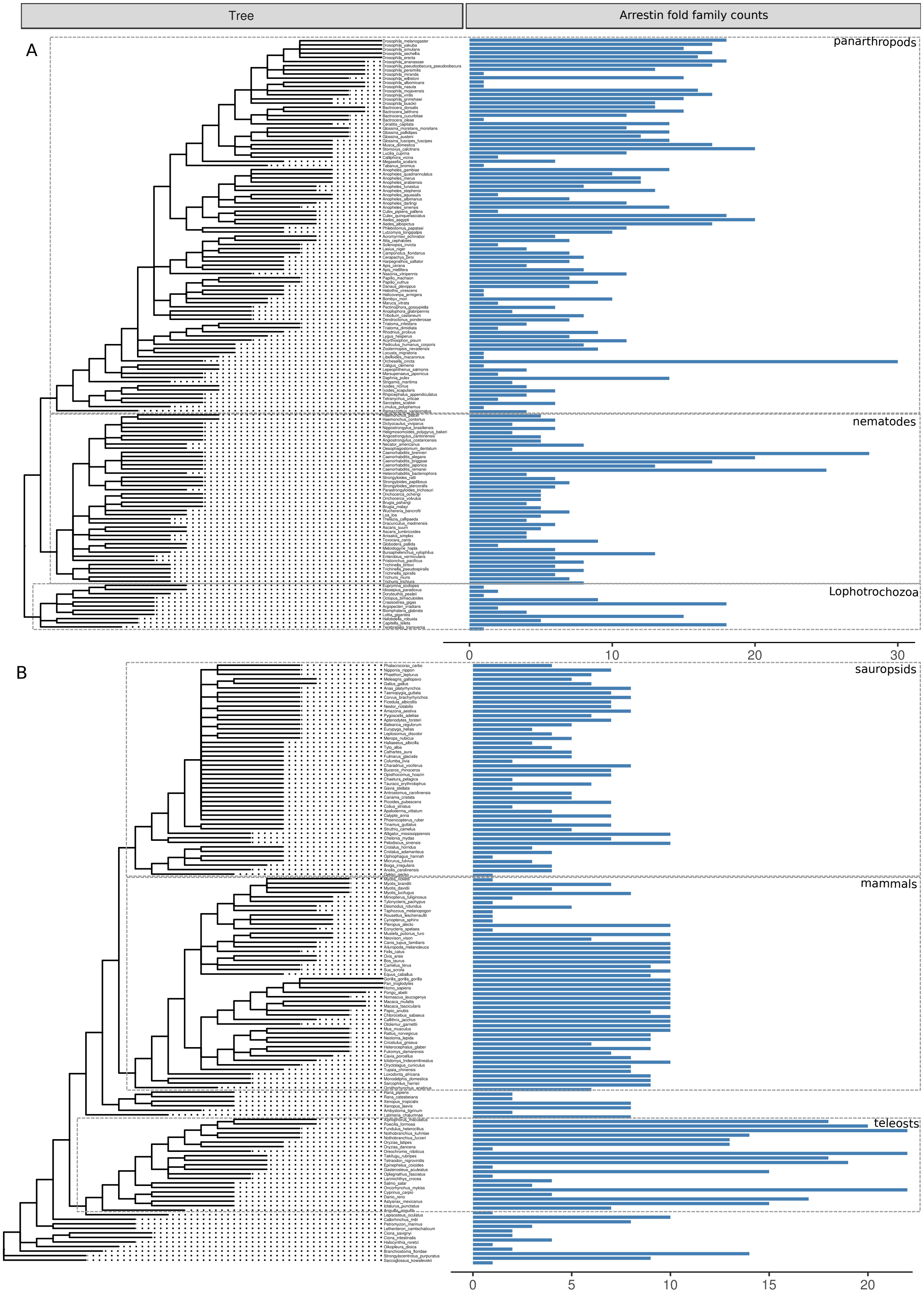

Figure S4 Abundance of arrestin fold family members in Bilateria according to `UniProtKB`**.** Hits were assigned to the arrestin fold family if they contained either an arrestin_N or an arrestin_C domain (see Methods) and were mapped to the NCBI taxonomy of protostomes (A) and deuterostomes (B).

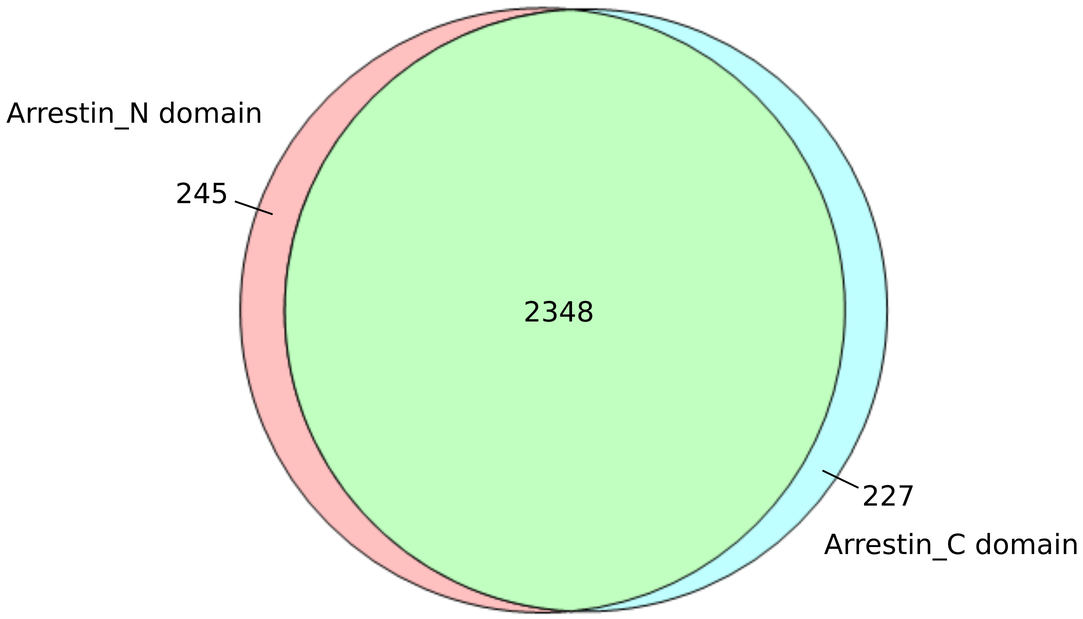

Figure S5 Scan of the `UniProtKB` with full-length arrestin pHMMs employing `jackhmmer`. `Jackhmmer` results after three iterations were filtered according to parameters specified in the Methods section resulting in 2962 hits in total. Those hits show a good overlap with the Pfam arrestin_N and arrestin_C domains (intersection, light green). The hits that did not contain any of the two domains (142) were excluded for estimation of paralog numbers and are not shown.

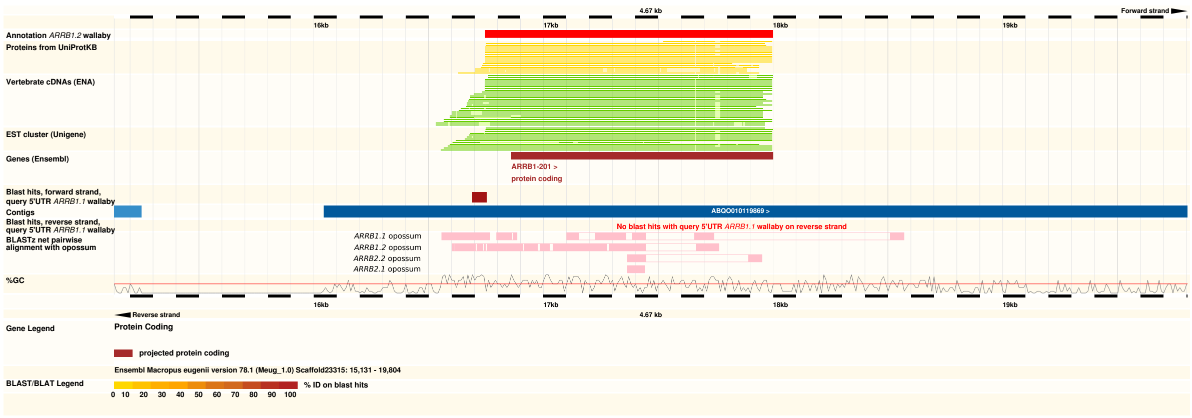

Figure S6 Structure and genomic locus of the ARRB1.2 retrogene in wallaby. The first row shows the genomic position of the coding sequence of the *ARRB1.2* gene as proposed in this study (bright red). It is in good accordance with arrestin sequences from `UniProtKB` (yellow), vertebrate cDNAs from `ENA` (green), and expressed sequence tag (`EST`) clusters from `Unigene` (green). In addition a fraction of cDNAs and EST clusters also show high sequence conservation to the region upstream of the proposed coding sequence. The "Gene (Ensembl)" track (dark red), denotes the protein coding gene *ARRB1-201*. Light pink boxes show high scoring `BLASTz` hits of the opossum arrestin loci against the *ARRB1.2* locus in wallaby. Notice that *ARRB1.2* of opossum likely is a retrogene sharing the retrotransposition event with wallaby. The figure was generated with the `Ensembl genome browser`. ID - identity; kb - kilobases.

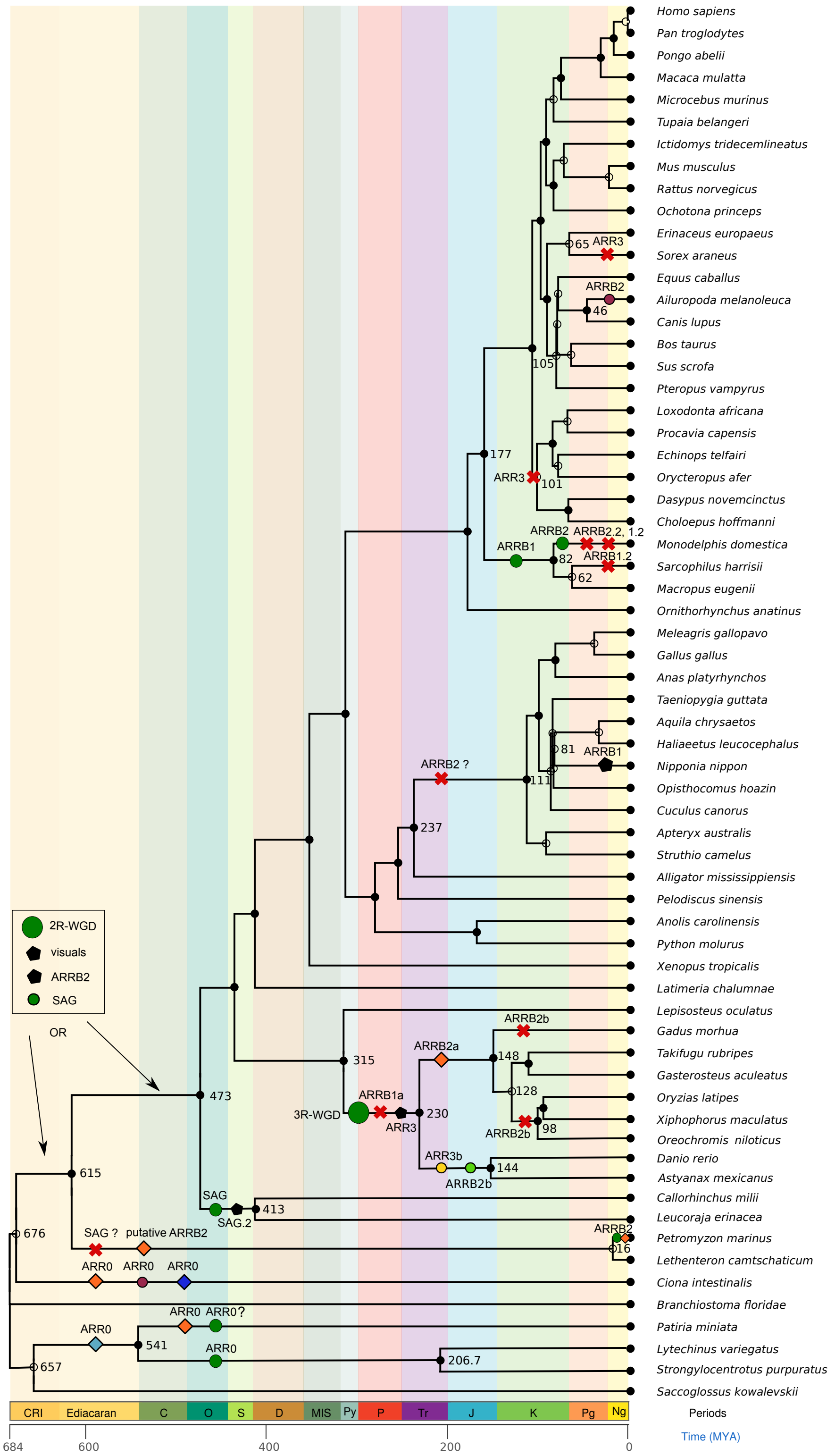

Figure S7 Summary of arrestin gene, exon and intron gain and loss events in deuterostomes. All events as inferred in this study were mapped onto a timed species tree [2], which allows for estimation of the time frame when those events happened. Trifurcations are labeled with the estimated speciation time, whenever events happened on nearby branches. Crosses and dark green dots indicate gene duplication and loss events, while colored diamonds, dots and pentagons on tree branches symbolize intron gain, intron loss and exon loss events, respectively. Gene duplications are often accompanied by gene structure changes. Arrestin genes in marsupials duplicated by retrotransposition, which also resulted into change of the gene structure (loss of all introns). Please see Fig. 10 in the main document for details on changes of the gene structure as well as color code. Note that events on the specific branches are placed arbitrarily regarding order and exact timing. Insertion of intron 85c in bat star must have occurred before duplication of *ARR0*, which is assumed to have happened very recently. The figure was created using the timetree webserver. MYA - million years ago; WGD - whole genome duplication; CRI - Cryogenian; C - Cambrian; O - Ordovician; S - Silurian; D - Devonian; M - Mississipian; Ps - Pennsylvanian; P - Permian; Tr - Triassic; J - Jurassic; K - Cretaceous; Pg - Paleogene; Ng - Neogene.

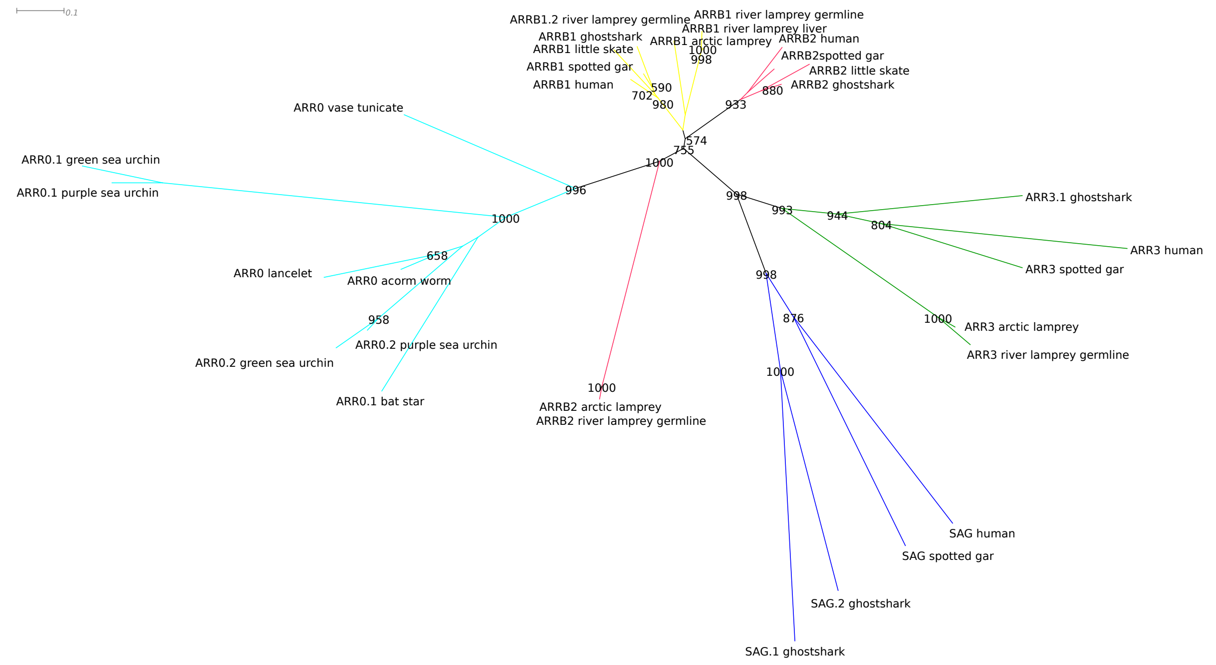

Figure S8 Maximum likelihood (ML) tree of arrestins from basal deuterostomes and human (lobe-finned fish) and spotted gar, a representative of bony fish. The tree was constructed from an amino acid alignment using `PhyML` (model LG+G with α 0.77 and 1000 bootstraps). The different monophyletic and well-supported orthology groups are highlighted in different colors. Bootstrap values are shown if support was 50..100%. The phylogenetic tree was visualized with `Dendroscope 3.5.7` [3].

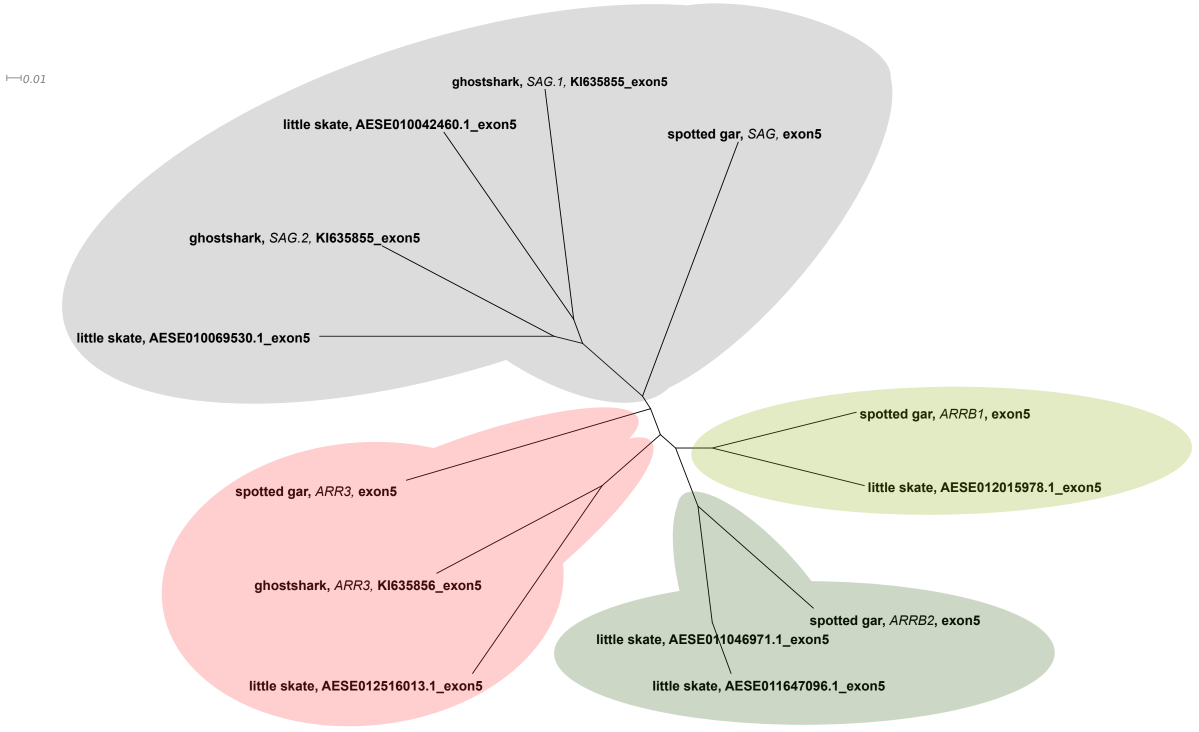

Figure S9 Neighbor-joining tree of exon 5 sequences from arrestins of spotted gar, ghost shark and little skate. As little skate has an extremely fragmented genome, the nucleotide sequence of the longest exon, i.e. exon 5, was used to build a phylogenetic tree of arrestins. In little skate, five full-length and one partial exon were detected. The tree was constructed using the neighbor joining clustering within `ClustalW 2.0.12`. The four orthology groups are clearly visible. Generally, little skate sequences (`AESE01xxxxxxx`) cluster with ghost shark sequences. Concerning the *SAG* paralogs (grey cloud), two distinct *SAG* genes exist, in ghost shark and little skate, suggesting a shared *SAG* gene duplication in the common ancestor. Exons of non-visual arrestins (green clouds) clearly cluster together, splitting in *ARRB1*s (light green) and *ARRB2*s (dark green). It is not clear, whether little skate possesses two *ARRB2*s, as exon 5 on `AESE01104697.1` is partial and the encoded part is 92.9% identical to exon 5 on `AESE011647096.1`. The phylogenetic tree was visualized with `Dendroscope 3.5.7` [3].

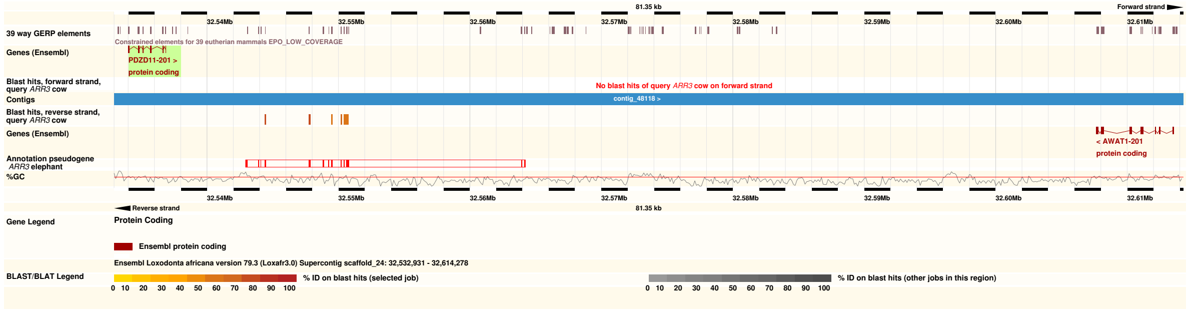

Figure S10 Structure and genomic locus of the ARR3 pseudogene in elephant. The *ARR3* pseudogene (bright red) is located between the `Ensembl` genes *PDZD11* and *AWAT1*. Fragments of the gene are still highly similar (*viz.* 50-60% identity) to bovine *ARR3* (track 1 and track 5). The picture was generated with the `Ensembl genome browser`. GERP elements - constrained, conserved elements called by `Ensembl`; ID - identity; kb - kilobases.

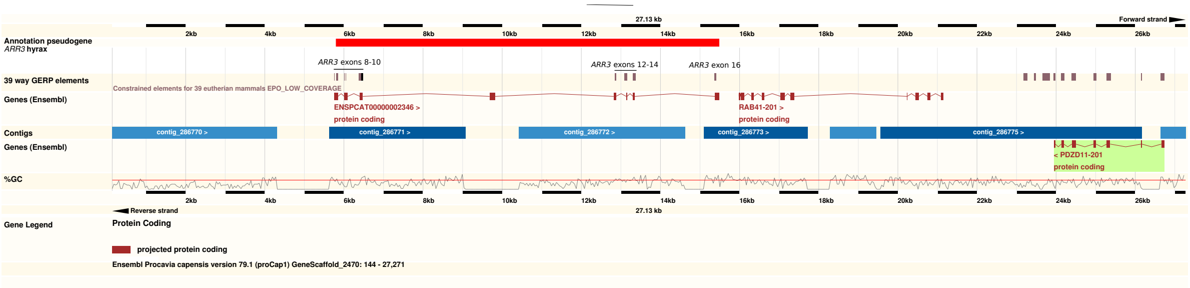

Figure S11 Genomic locus of the ARR3 pseudogene in hyrax. The respective locus was identified by investigation of the *PDZD11* (green box) neighborhood. No blast hits were retrieved with *ARR3* from cow as query. Nevertheless, there is sequence similarity to exons 8-10, exon 12-14 and exon 16 as indicated by high GERP conservation scores that point to *ARR3* in different eutherian mammals. The novel-protein coding gene ENSPCAT00000002346 that is annotated by Ensembl at this locus has an arrestin_C domain (PF02752). Besides the missing arrestin_N domain, attempts to annotate the C terminal part of arrestin in this region with ProSplign results in an annotation with two stop codons within exons, a frame shift and the need to annotate several non-canonical splice sites. The whole respective region is marked in bright red. The picture was generated with the Ensembl genome browser. GERP elements - constrained, conserved elements called by Ensembl; ID - identity; kb - kilobases.

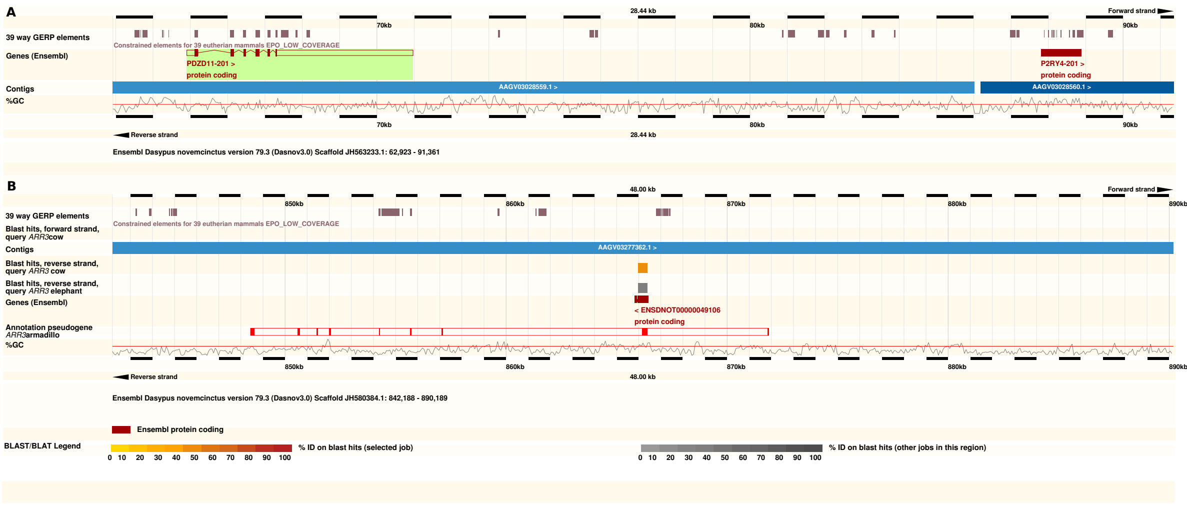

Figure S12 Candidate loci and genes for ARR3 in armadillo. A - The genomic region between *PDZD11* (green box) and *P2RY4* of armadillo has no similarity to bovine *ARR3*. B - Instead, bovine *ARR3* returned a blast hit on JH580384.1 overlapping the Ensembl gene ENSDNOT00000049106 that has an arrestin_N domain (PF00339). Annotation attempts using ProSplign resulted in annotation of a hypothetical pseudogene (bright red) that contains 3 internal stop codons and 3 frame shifts. The picture was generated with the Ensembl genome browser. GERP elements - constrained, conserved elements called by Ensembl; ID - identity; kb - kilobases.

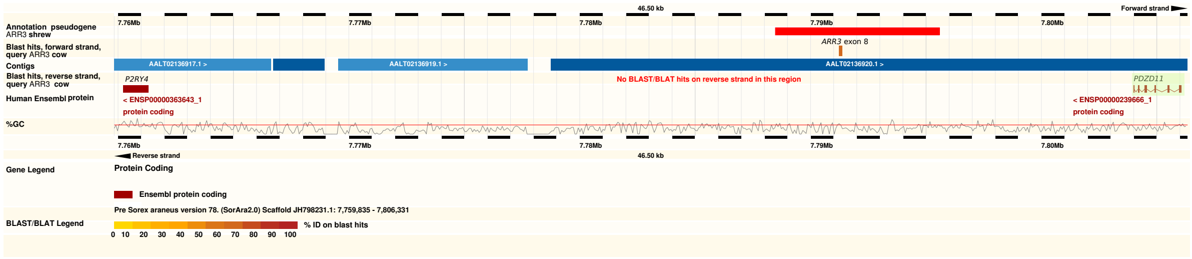

Figure S13 Genomic locus of the ARR3 pseudogene in shrew. The respective locus next to *PDZD11* was identified by `tblastn` of bovine *ARR3* against the shrew genome (green box). Homology search using *ARR3* from dog, human and mouse revealed fragments similar to exons 3, 8, 10, 12 and 14. Attempts to annotate the full coding sequence with `ProSplign` resulted in an annotation with at least five internal stop codons. The region spanning the putative exons 3-14 is therefore proposed to represent an *ARR3* pseudogene (bright red). Note that the contig is ungapped in this region. The picture was generated with the `Pre!Ensembl genome browser`. ID - identity; kb - kilobases.

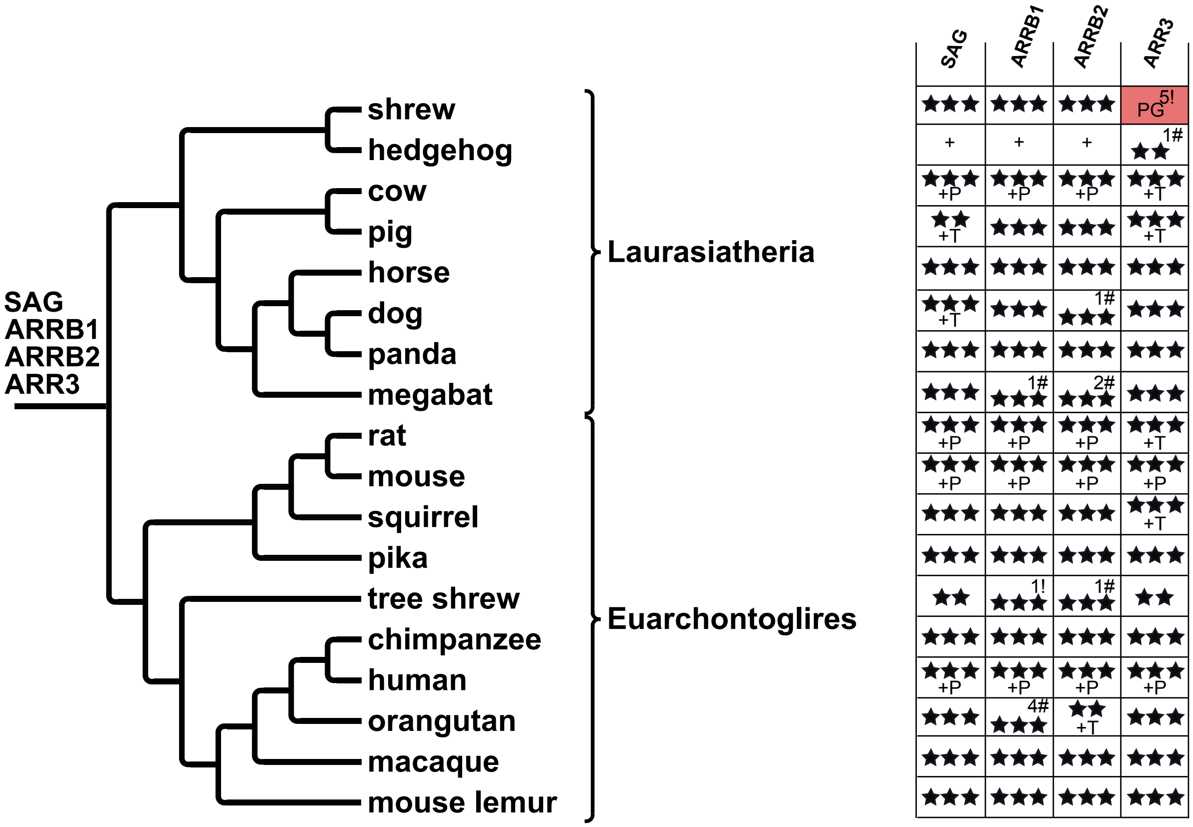

Figure S14 Arrestin paralogs within Laurasiatheria and Euarchontoglires. Four arrestin paralogs are encoded in the genomes of both mammalian clades with one exception, *ARR3* in shrew. The gene is probably degraded to a pseudogene (red box). It is not clear, whether this is also true for hedgehog, which has a highly fragmented *ARR3* locus likely due to missing data. The table on the right side of the figure depicts the completeness of arrestin annotation in the respective genomes. Additional support for arrestins from reviewed entries of `UniProtKB` are given in the table. See caption of Fig. 3, 6 in the main document for additional description of symbols. The phylogenetic tree was created with `Treegraph 2.0.54` [4].

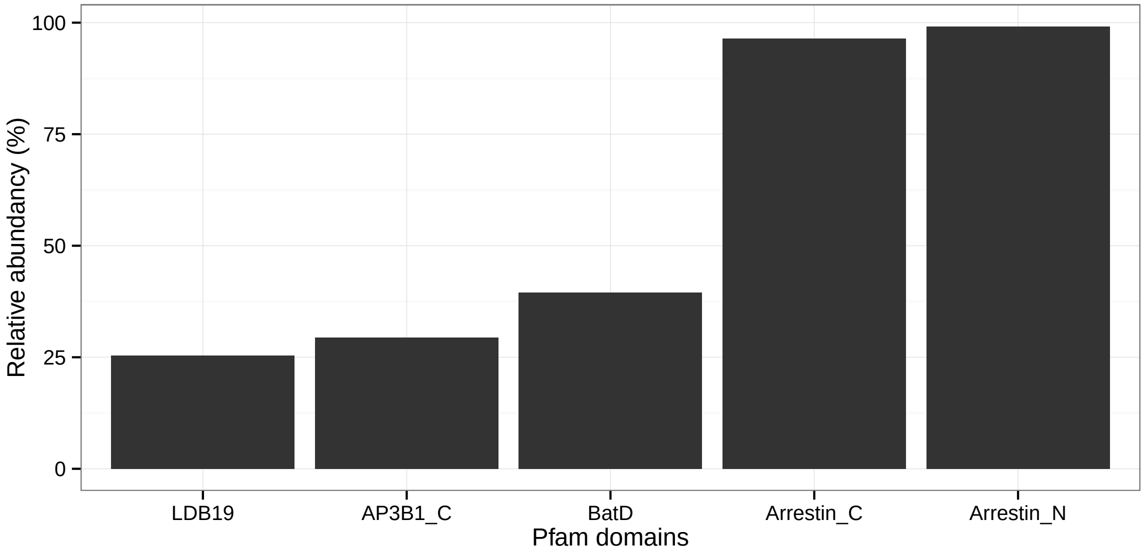

Figure S15 **`Pfam` domains in deuterostome arrestins.** All deuterostome arrestins (excluding pseudogenes and fragments of *ARRB2* in birds) were scanned against the `Pfam 28.0` database [5]. The relative abundance of domains, present in at least 25% of all deuterostome arrestins, is shown.

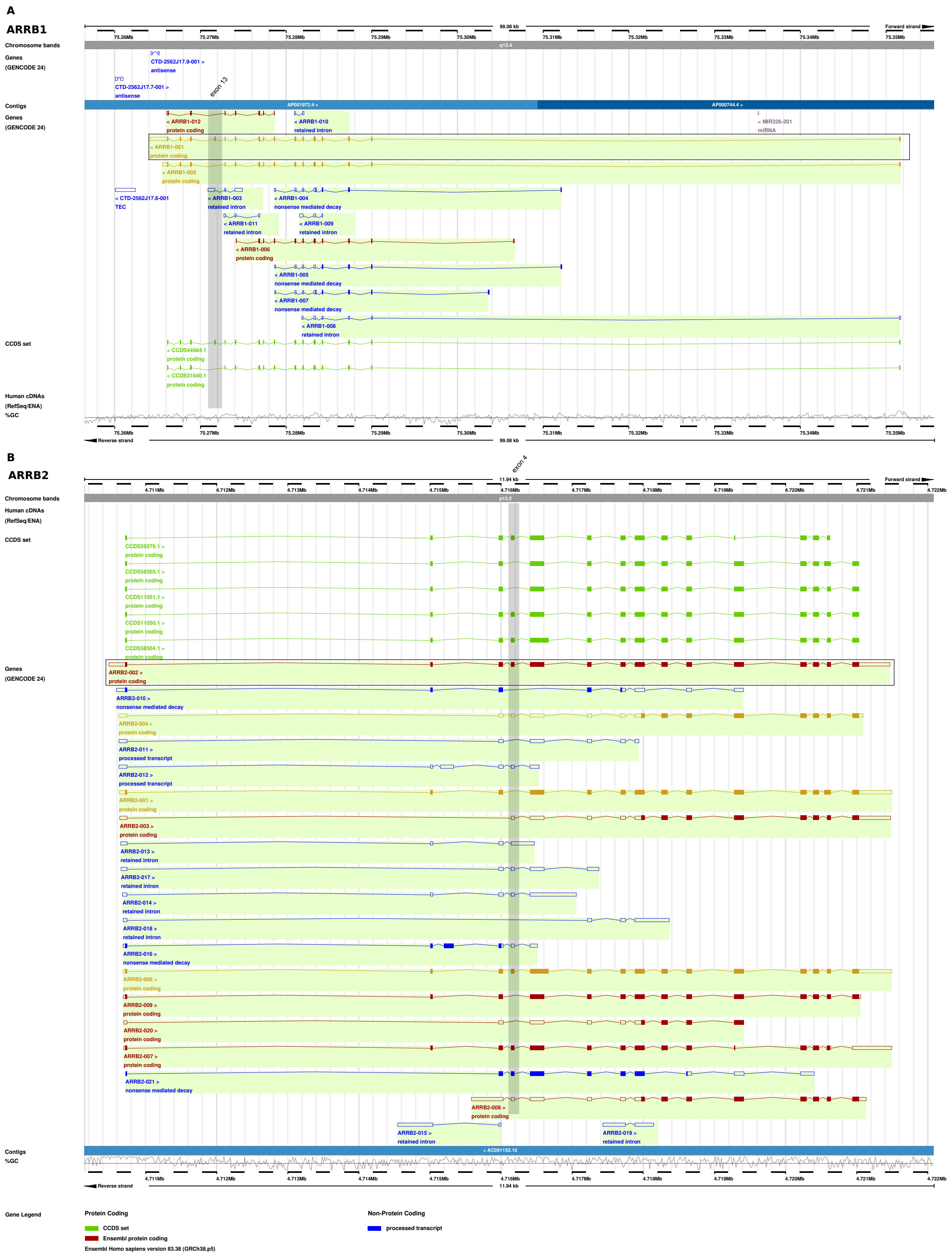

Figure S16 Gene structure and alternative transcript annotations of non-visual arrestins in human used to derive deuterostome arrestin sequences. A - Genomic region on chromosome 11 showing the *ARRB1* locus in human. B - Genomic region on chromosome 17 showing the *ARRB2* locus in human. The coding sequences of *ARRB1* and *ARRB2*, used in this study, are retrieved from the transcripts *ARRB1-001* and *ARRB2-002* (black boxes), respectively. Exons 13 and 4 were investigated for splice site conservation in deuterostomes (highlighted by grey shaded boxes). Note that the exon numbering refers to protein-coding exons. The figure was generated with the `Ensembl genome browser`, genome version GRCh38.p5. CCDS - consensus coding DNA sequence; kb - kilobases.

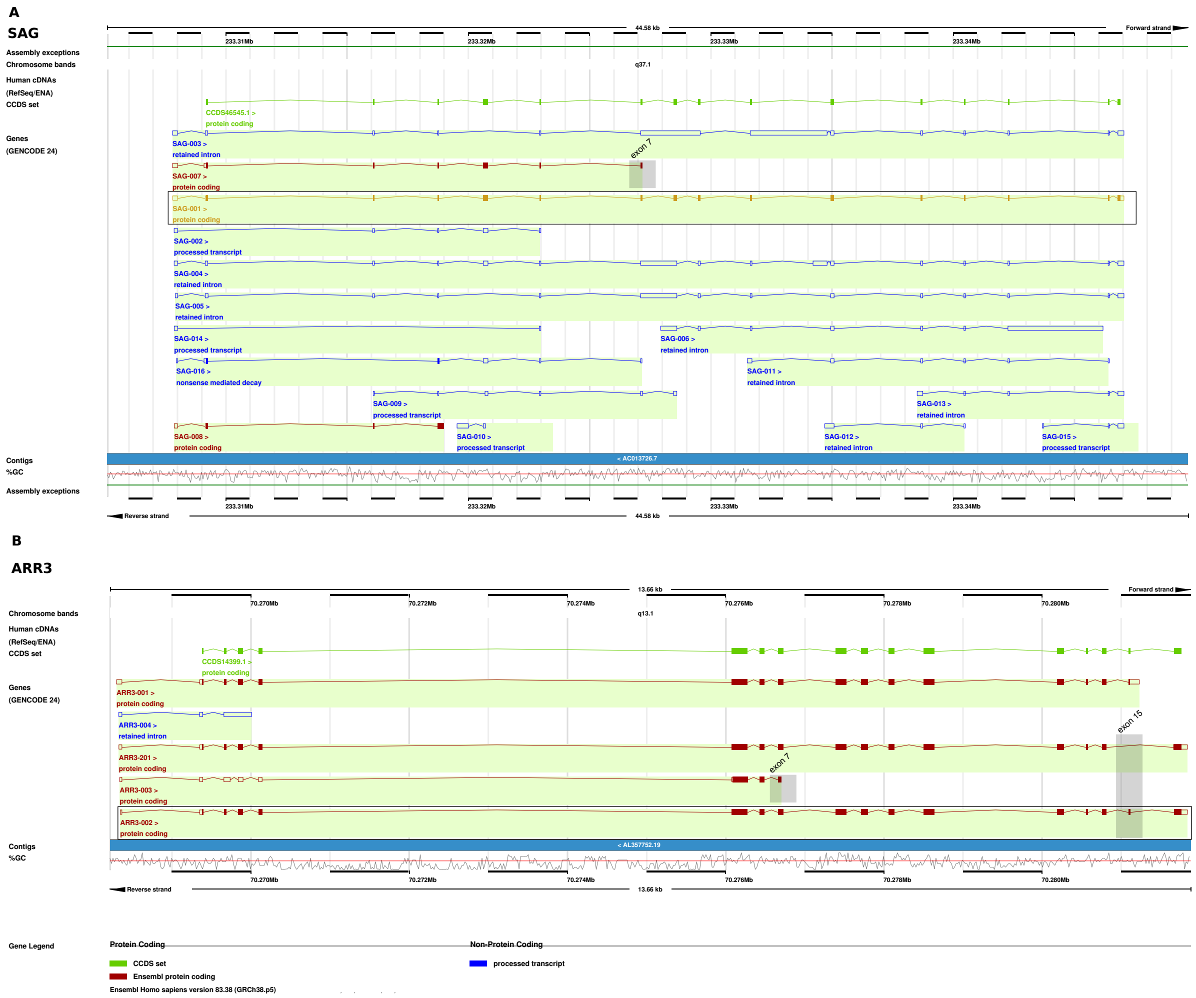

Figure S17 Gene structure and alternative transcript annotations of visual arrestins in human used to derive deuterostome arrestin sequences. A - Genomic region on chromosome 2 showing the *SAG* locus in human. B - Genomic region on chromosome X showing the *ARR3* locus in human. The coding sequences of *SAG* and *ARR3*, used in this study, are retrieved from the longest transcripts, *SAG-001* and *ARR3-002* (black boxes), respectively. Exon 15 and exon 7 were investigated in deuterostomes for conservation of splice sites and an encoded stop codon, respectively (highlighted by grey shaded boxes). Note that the exon numbering refers to protein-coding exons. The figure was generated with the `Ensembl genome browser`, genome version GRCh38.p5. CCDS - consensus coding DNA sequence; kb - kilobases.

Appendix 1 — Arrestin inventories in lampreys

While two non-visual arrestins were annotated without difficulty in the arctic lamprey, annotation of visual arrestins turned out to be problematic. The putative locus of *ARR3* was extremely fragmented with 12 exons situated on six different contigs. Nevertheless, predictions were consistent with the results of [6], who cloned one non-visual arrestin and one visual arrestin from arctic lamprey's pineal organ. Investigation of the germline genome of river lamprey retrieved these three 1:1 orthologs as well as a river lamprey specific non-visual paralog (Fig. 8, Fig. 4 in main document). This arrestin is most similar to *ARRB1* and might have arisen from an independent gene duplication event. The sequence of the other non-visual arrestin corresponds to the single arrestin gene that was detected in the liver tissue genome of the same species. The apparent discrepancy in the number of arrestin paralogs in germline and liver tissue of river lamprey might be ascribed to programmed loss of germline DNA in somatic cells, which has been postulated to pertain 20% of the germline DNA including protein-coding DNA [7].

Apart from those paralogs, we detected some more exons in the germline genomes of both lamprey species that seem to belong to arrestins and share highest similarity to exons of non-visual arrestins. We could not identify any exons orthologous to *SAG* in either lamprey genome unambiguously. Thus, the arrestin inventory for lampreys remains incomplete.

Appendix 2 — Annotation of arrestins in cartilaginous fish

In the ghost shark genome, only three exons of non-visual arrestins were detected. To verify their existence, we complemented the genomic data by searching the `NCBI Expressed Sequence Tag` (`EST`) and `NCBI transcriptome shotgun assembly` (`TSA`) database for cartilaginous fish. We identified homologs of both *ARRB1* and *ARRB2* for two species of Elasmobranchii (catshark, little skate) and for one species of Chimaera (ghost shark).

In order to elucidate, whether the duplication of *SAG* is chimaera-specific or shared with Elasmobranchii, the highly fragmented genome (26x) of the little skate was also investigated. Within this genome, only fragments of arrestins (1-4 exons) were found to be situated on the same genomic fragment. As the exon-intron structure is highly conserved among vertebrate arrestin paralogs, the number of confident, but different hits of one exon-family of sufficient length can be taken as an estimate for the number of paralogs within the species. In little skate, five complete and one partial exon 5 were detected with E-values $<= 1.4e-05$, whereby the incomplete exon 5 was located at the end of contig `AESE011046971.1`. The observation of at least five paralogs is further supported by the detection of five reliable sequences for exons 3 and 9 each (E-values $<= 4.4e-07$ and $<= 1.9e-04$, respectively). To finally confirm that the additional paralog is in fact a second *SAG* paralog, a neighbor joining tree of exon 5 from little skate, spotted gar and ghost shark was constructed on nucleotide level using `ClustalW 2.0.12` [8]. As expected, one exon 5 sequence from little skate clustered together with *SAG.1* and *SAG.2* from ghost shark respectively, forming a monophyletic group with *SAG* from spotted gar (Fig. 9).

Appendix 3 — Evolution of visual arrestins in different orders of teleosts

As apparent from inspection of the multiple correspondence analysis (MCA), *SAGb* and *ARR3b* of different teleost orders show systematic differences within their respective monophyletic groups.

Visual inspection of the MCA shows that Otomorpha *SAGb*s form a sub-group within teleost *SAGb*. This subdivision in Otomorpha and Euteleosteomorpha is also apparent upon inspection of the low affinity IP6 binding site (Fig. 7 A in main document). Here, the positively charged residue R167, which is part of that motif, was substituted by a neutrally or negatively charged amino acid in Euteleosteomorpha *SAGb* (E, Q, A) [9]. In Otomorpha *SAGb*, all *SAGa* and *SAG* of spotted gar, the positively charged arginine is conserved. A neighboring residue (165) was converted to arginine in the teleost *SAGa* stem lineage, while this position

is occupied by negatively or neutrally charged amino acids in *SAGb* (Q, C, D). This is further confirmed by the fact that 13% of sites of *SAGb* evolve under positive selection in the ancestral branch leading to the sister group Acanthopterygii (Euteleosteomorpha without cod; see Additional file 2).
Similarly to *SAGb*s, *ARR3b*s of Otomorpha cluster closer together than the other teleost *ARR3b*s. Concerning receptor binding residues, Euteleosteomorpha *ARR3b* show different patterns from all other teleost *ARR3* sequences (e.g. pos. 76, 246, 248, 254, Fig. 7 D in main document). 14% of *ARR3b*'s residues evolved under positive selection in the ancestral branch leading to Euteleosteomorpha (e.g. pos. 254, see Additional file 2). Differences in Euteleosteomorpha *ARR3b* are also apparent in phosphate sensing residues as *ARR3a* possesses one or two additional positive charges in the sequence stretch that mediates low affinity IP6 binding in *SAG* (K152 or K154 and K157, Fig. 7 C in main document, [9]). Otomorpha *ARR3b* have intermediate properties (conserved K157) and form a subgroup within teleost *ARR3b*. The low affinity IP6 binding site is conserved in all vertebrate *ARR3* otherwise, although IP6 binding has not been characterized yet experimentally.

Appendix 4 — Investigation of the *ARR3* locus in Afrotheria, Xenarthra and common shrew

Elephant

The elephant *ARR3* gene shares the exon-intron structure with its human arrestin ortholog. Fragments of the protein sequence show 61% identity to the human ortholog (Fig. 10). All other placental *ARR3* share a sequence identity of more than 80% with the human and horse *ARR3* translation product. In human, the genes *P2RY4* and *AWAT1* are situated upstream of the *ARR3* gene, and *RAB41* and *PDZD11* downstream, respectively. Synteny is conserved in comparison to other mammals with the genes *AWAT1* and *PDZD11* located upstream and downstream of the *ARR3* pseudogene in elephant, respectively. Even under the assumption of non-canonical splice sites, the best annotation of elephant *ARR3* encodes for six stop codons within the putative protein-coding sequence. It is thus unlikely that the gene codes for a functional full-length arrestin. If so, additionally, mutations in key functional elements occurred e.g. in the polar core (D297Y) or in residues important for receptor specificity (C282F, T259/261).

Hyrax

In contrast to elephant, sequence in between *AWAT1* and *PDZD11* is not completely covered in the genome of hyrax (Fig. 11). Nevertheless, some sequence clearly shows similarity to exons 8-10, 12, 14 and 16 of *ARR3* with 26% identity to the human *ARR3* query. Although sequence in between exons 12 and 14 is completely sequenced, exon 13 cannot be identified by homology search. In conclusion, this points at a degradation of *ARR3* to pseudogenes in both investigated Paenungulata genomes, elephant and hyrax.

Armadillo

In armadillo, *P2RY4* and *PDZD11* are situated on the same contig, `JH563233`, about 16.5 kb apart (Fig. 12 A). The loss of *ARR3* in armadillo is supported by two facts: (1) The single blast hit that was obtained for this locus with the nucleotide sequence of the elephant *ARR3* pseudogene and the bovine *ARR3* gene as queries, has a low similarity towards the queries (Fig. 12 A). (2) The locus between *P2RY4* and *PDZD11* is shortened in comparison to the length of the *ARR3* gene in mammals (e.g. 22 kb in human). The `tblastn` search against the whole genome of armadillo with *ARR3* from cow as query retrieved one hit that did not overlap with other annotated arrestin loci (*E*-value 1e-1), but with the novel protein-coding gene `ENSDNOT00000049106`. This gene was annotated by the `Ensembl` gene prediction pipeline and has the arrestin_N domain (PF00339) (Fig. 12 B). Nevertheless, the locus can be excluded as (1) the exon-intron structure is not conserved in comparison to other placental *ARR3*, (2) annotation of a stop codon and several

frame shifts would be necessary, (3) sequence identity to *ARR3* in horse is extremely low with 36%.

Others

In the three other investigated xenarthran and afrotherian genomes, the neighboring genes are either situated on different genomic fragments (sloth and aardvark) or are lost to Ns (tenrec). No hits were retrieved for *ARR3* in the genomes of aardvark, tenrec and sloth. An independent degradation of *ARR3* was detected in the genome of common shrew (Fig. 13, 14). The region between the genes *PDZD11* and *P2RY4* contains fragments that have some similarity to exons 3, 8, 10, 12 and 14 of *ARR3* of other mammals. Annotation with `ProSplign` retrieved a degraded gene that encodes for at least five stop codons within exons, has no start and stop codon. While exons 1 and 2 could possibly be situated in a region of Ns, the stretch between fragments of exons 3-14 is fully encoded supporting the annotation as a pseudogene.

Appendix 5 — Investigation of loss of *ARRB2* in Sauropsids

In order to assign exons to arrestin-3 (*ARRB2* gene) within birds, complete amino acid sequences of the four paralogs of close relatives were each blasted against the respective bird genomes using `blastall 2.2.26` with the `-p tblastn` option. Contigs and scaffolds will be called genomic fragments in the following. The hits were sorted by E-value and the genomic fragments were assigned to the paralog, that obtained the highest scoring blast hit on that genomic fragment. Paralogs were checked for completeness. Usually, *SAG*, *ARR3* and *ARRB1* were almost completely situated on one genomic fragment and included in the respective annotation. All hits that were not assigned to any of the other three arrestins regardless of which arrestin protein query retrieved the hit, were inspected again manually to clarify whether they belonged to *ARRB2* (Additional file 2, bird annotations).

Additionally, the nucleotide sequence of *ARRB2* exons identified in the kiwi genome was blasted against the `NCBI short read archive` (`SRA`) of close relatives, ostrich and tinamou. The same was repeated for the `SRA` of gold eagle and white-tailed eagle with bald eagle *ARRB2* exons as query. This approach retrieved exons that were not recovered from the assembled genomes with `tblastn` otherwise, pointing to problems with the assembly of these bird genomes.

As evidence for *ARRB2* was very sparse after conventional homology search on genomic level, the approach was extended to include: (1) homology search for genes assumed to be neighbors of *ARRB2* (i.e. synteny information) to detect cases of loss and pseudogenization events and, (2) homology search for *ARRB2* in available bird transcriptome/`EST` data. First, syntenic information had to be inferred from the *ARRB2* locus in other species. Whenever synteny information was available for the investigated mammalian genomes, *Med11* and *Pelp1* were found to be neighbors of *ARRB2*, oriented head to tail of *ARRB2* and head to head of *ARRB2*, respectively. Within sauropsids (birds and reptiles) the head to tail neighborhood to *Med11* is supported by alligator, while the head to head neighborhood to *Pelp1* is supported by genomic information from turtle, python and frog. In most other cases, synteny information was not available due to a low assembly/sequencing status of the respective genomes. Furthermore, *Med11* was also found next to *ARRB2* in the genome of coelacanth, the outgroup to sauropsids and mammals. This suggests that *ARRB2* was located between *Med11* and *Pelp1* in the last common ancestor of lobe-finned fish and a conservation of this linkage throughout lobe-finned fish. In this study, only *ARRB2* in frog was found to have the gene *DDX27* as a neighbor in place of *Med11*. The latter was found in a completely different gene neighborhood, which might be the result of an amphibian-specific rearrangement. None of the potential neighboring genes, *Med11* or *Pelp1*, was detected in the genomes of the investigated bird species or in lizard.

Second, the genome-focused approach was complemented using specific bird transcriptome data sets (Table 2). Data from three sources for zebrafinch and chicken and the `EST` and `TSA` database of birds, were queried

with known *ARRB2*s. Whole or partial hits were retrieved for *SAG*, *ARRB1* or *ARR3*, while in general no hits were retrieved for *ARRB2*. Within the investigated chicken ovary expression data [10], some fragments were recovered that could not be assigned to neither *ARRB1* nor *ARRB2* unambiguously, but were similar to a non-visual arrestin.

Appendix 6 — Domains of deuterostome arrestins

Apart from the arrestin_C and arrestin_N domains, the following other domains were detected in more than 25% of the deuterostome arrestins: BatD, a membrane spanning protein connected to oxygen tolerance in bacteria, the clathrin-adapter complex 3 beta 1 subunit C terminal domain (AP3B1_C) and the arrestin_N terminal like domain (LDB19), which belongs to the arrestin N-like clan (Fig. 15). The domains were not specific for certain orthology groups. For AP3B1_C, all obtained hits had a conditional E-value $<$ 9.4e-05 and covered 19-47% of the profile. Mapped onto arrestins, the domain overlapped with the beginning of the arrestin_C domain and covered residues that are known to be involved in microtubule, calmodulin and phosphodiesterase binding (residues 192-237 in bovine *ARRB1*). AP3B1 is part of the adapter protein-complex and interacts with clathrin via a clathrin binding motif as does arrestin.

Appendix 7 — Isoforms and changes of the conserved exon-intron structure

Conservation of cassette exons and short isoforms was investigated, if this behavior appeared systematically in different isoforms of the same paralog (skipping of exon 13 in *ARRB1*, skipping of exon 4 in *ARRB2*) or across paralogs (stop after exon 7 in *SAG* and *ARR3*) as annotated in the `Ensembl genome browser` for human (Fig. 16, 17). Skipping of exon 15 as seen in *ARR3* was additionally considered as this exon encodes the major clathrin binding site. All results are also available in tabular format in Additional file 2, Possible isoforms.

Skipping of exon 15

In both visual arrestins, exon 15 is extremely shortened to only 10 to 16 nt in comparison to exon 15 of *ARRB1* which has a length of 51 nt in human and contains the major clathrin binding site. Despite variation in the length of exon 15, the extension of exon 14 to the next downstream canonical splice site allows skipping of exon 15 under preservation of the reading frame, with the exception of *SAG* in fish and *ARR3* in coelacanth. The other visual arrestin, *ARR3*, in fish showed loss of exon 15/16 supported by the `Ensembl` gene annotation and mRNA sequence [11]. However, presence or absence of exon 15 remains unresolved for some mammals. This is due to the fact that exon 15 is extremely short and can show high sequence variation impeding detection by homology search.

Stop after exon 7

`EST` data from `Ensembl` for *SAG* and *ARR3* supported the existence of an extremely shortened isoform in human (Fig. 17). In this isoform, exon 7 is extended into intron 161b towards an encoded stop codon. The genomic data showed that this stop codon downstream of exon 7 is conserved in *SAG* of all deuterostomes, in mammalian and sauropsid *ARRB1* and in mammalian *ARRB2*. It is only loosely conserved in fish *ARRB1* and *ARRB2*. Unexpectedly, for *ARR3*, the stop codon shows a dispersed absence/presence pattern in these three clades. As the premature stop is conserved in *ARR0* of lancelet and vase tunicate, this stop codon was acquired during early chordate evolution with subsequent losses and re-acquisitions in specific classes.

Skipping of exon 4 and exon 13

Exons 4 and 13 are spliced out in specific isoforms of *ARRB2* and *ARRB1* in human, respectively. The length of exons 4 and 13 on nucleotide level and the respective reading frames are conserved in all paralogs suggesting that these exons could be skipped in all investigated chordates. An exception is exon 15 in the river lamprey specific non-visual arrestin. Exon 4 has several residues contributing to receptor binding and specificity. Interestingly, exon 4 is fused to exon 5 in all echinoderms and hemichordates precluding this isoform.

Deviations in the conserved exon-intron structure

In general, all paralogs have a highly conserved exon-intron structure with only few exceptions. Apart from the exceptions mentioned above, these changes concern the fusion of exons 14 and 15 in *ARRB2* from panda, and the loss of exon 13 in *ARRB1* from ibis (Fig. 10 B in main document). As sequence data is fully available in the intronic region between exons 12 and 14 in ibis, exon 13 and thus the minor clathrin binding site is probably lost in this bird. Within Euteleosteomorpha, exon 5 of *ARRB2a* is split into two exons, while exons 6 and 7 are fused in Otomorpha *ARRB2b*. All duplicated fish lost exon 16 of *ARR3* and possess a shortened exon 15. Conceptional translation thus results in a C-terminally shortened *ARR3*. Furthermore, exons 12 and 13 of Otomorpha *ARR3b* are fused resulting in a gene structure with 14 protein-coding exons. No homolog of exon 16 could be detected for ghost shark *SAG.2* hinting at a loss of exon 16.

Appendix 8 — Parsimonious reconstruction of intron gain/loss events

Exons are numbered according to human *ARRB1* with homologous exons sharing the same number (see Fig. 10 A in main document). A maximum parsimony reconstruction of intron loss and gain events points to hotspots at positions 85c (five independent intron gains), at 138c (three independent events), 235a (two independent events) and 365a (two independent intron losses) (Fig. 10 B, C in main document). All other changes probably represent single events of intron gain or loss. As the above mentioned events must have taken place several times, several scenarios exist with the same number of events (intron gains and losses). We minimize the number of events without resolving whether these are actually intron gains or losses considering the ongoing and unresolved discussion about introns-late vs. introns-early concepts [12]. For counting the number of events, the root state was hypothesized to be the same as in fruit fly's phosrestin-1 (*Drosophila melanogaster*) and roundworm arrestin (*Caenorhabditis elegans*), which have no introns at the named hotspots, with exception of intron 138c in roundworm.

Appendix 9 — Annotation of arrestins in mammals

We started annotation of arrestin homologs based on the arrestin reference sequences in `UniProtKB`. These correspond to the well characterized and on transcriptome level supported annotations of the longest isoforms of three of the four arrestin paralogs in human retrieved from `Ensembl` ([13], Fig. 16, 17). First, annotation of arrestin homologs in 13 different mammalian orders were systematically completed. Second, an initial alignment was built from these sequences. To do so, query protein sequences were blasted against the respective genome of interest using `tblastn` on the `Ensembl` web interface [14]. Missing short exons were retrieved using local `tblastn` or `blastn` (`bl2seq 2.2.26`, E-value < 1) and the spliced alignment tool `ProSplign` [15]. The reference sequence for *ARRB2* (409 AA) does not contain the 22 AA extension of exon 5 seen in the longest isoform in human ([13], Fig. 16).

Table S1: **List of genomes used for the current study.** Latin and trivial names are provided together with the version used and source of investigated assemblies. Additionally, all other 39 genomes from the Avian genomics project were investigated.

Latin name	Trivial name	Genome version	Genome source
Homo sapiens	human	GRCh38	Ensembl
Monodelphis domestica	opossum	monDom5	Ensembl
Tupaia belangeri	tree shrew	tupBel1	Ensembl
Ictidomys tridecemlineatus	squirrel	spetri2	Ensembl
Erinaceus europaeus	hedgehog	eriEur1	Ensembl
Loxodonta africana	elephant	Loxafr3.0	Ensembl
Dasypus novemcinctus	armadillo	Dasnov3.0	Ensembl
Ornithorhynchus anatinus	platypus	OANA5	Ensembl
Pongo abelii	orangutan	PPYG2	Ensembl
Pan troglodytes	chimpanzee	CHIMP2.1.4	Ensembl
Equus caballus	horse	Equ Cab 2	Ensembl
Sarcophilus harrisii	Tasmanian devil	Devil_ref v7.0	Ensembl
Procavia capensis	hyrax	proCap1	Ensembl
Echinops telfairi	tenrec	TENREC	Ensembl
Bos taurus	cow	UMD3.1	Ensembl
Sus scrofa	pig	Sscrofa10.2	Ensembl
Pteropus vampyrus	megabat	pteVam1	Ensembl
Ailuropoda melanoleuca	panda	ailMel1	Ensembl
Macaca mulatta	macaque	MMUL 1.0	Ensembl
Choloepus hoffmanni	sloth	choHof1	Ensembl
Orycteropus afer afer	aardvark	OryAfe1.0	Pre!Ensembl/GCA_000298275.1
Macropus eugenii	wallaby	Meug_1.0	Ensembl
Sorex araneus	shrew	sorAra2.0	Pre!Ensembl/GCA_000181275.2
Microcebus murinus	mouse lemur	micMur1	Ensembl
Mus musculus	mouse	GRCm38.p1	Ensembl
Rattus norvegicus	rat	Rnor_5.0	Ensembl
Ochotona princeps	pika	OchPri3	Pre!Ensembl/GCA_000292845
Canis familiaris	dog	CanFam3.1	Ensembl
Meleagris gallopavo	turkey	Turkey_2.01	Ensembl
Meleagris gallopavo	turkey	Turkey_5.0	NCBI/GCF_000146615.2
Anas platyrhynchos	duck	BGI_duck_1.0	Ensembl
Taeniopygia guttata	zebra finch	taeGut3.2.4	Ensembl
Aquila chrysaetos	golden eagle	v1.0.2	NCBI/GCF_000766835.1
Apteryx australis mantelli	kiwi	v1.0	NCBI/GCF_001039765.1
Anolis carolinensis	anole lizard	AnoCar2.0	Ensembl
Xenopus tropicalis	frog	JGI 4.2	Ensembl
Pelodiscus sinensis	turtle	PelSin_1.0	Ensembl
Gallus gallus	chicken	Galgal4	Ensembl

Continued on next page

Table S1– continued from previous page

Latin name	Trivial name	Genome version	Genome source
Latimeria chalumnae	coelacanth	LatCha1	Ensembl
Lepisosteus oculatus	spotted gar	LepOcu1	Ensembl
Python molurus bivittatus	python	Python 5.0.2	NCBI/GCA_000186305.2
Alligator mississippiensis	alligator	v0.1d27	ftp://ftp.crocgenomes.org/pub/ ICGWG/Genome_drafts/alligator.old /amiss_v0.1d27/amiss_v0.1d27.fa
Cuculus canorus	cuckoo	v1.0	http://avian.genomics.cn/en/jsp/ database.shtml
Haliaeetus leucocephalus	bald eagle	v1.0	http://avian.genomics.cn/en/jsp/ database.shtml
Opisthocomus hoazin	hoatzin	v1.0	http://avian.genomics.cn/en/jsp/ database.shtml
Nipponia nippon	ibis	v1.0	http://avian.genomics.cn/en/jsp/ database.shtml
Struthio camelus	ostrich	v1.0	NCBI/GCA_000698965.1
Gadus morhua	cod	gadMor1	Ensembl
Takifugu rubripes	pufferfish	FUGU4	Ensembl
Oreochromis niloticus	tilapia	Orenil1.0	Ensembl
Oryzias latipes	medaka	MEDAKA1	Ensembl
Xiphophorus maculatus	platyfish	Xipmac4.4.2	Ensembl
Gasterosteus aculeatus	stickleback	BROADS1	Ensembl
Astyanax mexicanus	cave fish	AstMex102	Ensembl
Danio rerio	zebrafish	Zv9	Ensembl
Callorhinchus milii	ghost shark	calMil1.fa	UCSC (calMil1.fa.gz)
Leucoraja erinacea	little skate	v1.0	NCBI/GCA_000238235.1
Ciona intestinalis	vase tunicate	JGI2	Ensembl
Petromyzon marinus	river lamprey	Pmarinus_7.0	Ensembl
		germline genome	personal communication (Chris Amemiya, April 2016)
Lethenteron camtschaticum	arctic lamprey	v1.0	NCBI/GCA_000466285.1
Branchiostoma floridae	lancelet	Brafl1_v2.0	http://genome.jgi-psf.org/ Brafl1/Brafl1.download.html Branchios- toma_floridae_v2.0.assembly.fasta.gz
Strongylocentrotus purpuratus	purple sea urchin	Spur_3.1	Ensembl Metazoa
Patiria miniata	bat star	v1.0	http://www.echinobase.org/ Echinobase/PmDownload (pmin.scaf.fa)
Lytechinus variegatus	green sea urchin	v0.4	http://www.echinobase.org/ Echinobase/ LvDownload (Lvar_0.4.20110428.linear.fa)

Continued on next page

Table S1– continued from previous page

Latin name	Trivial name	Genome version	Genome source
Saccoglossus kowalevskii	acorn worm	JGI3.0	ftp://ftp.jgi-psf.org/ pub/compgen/metazome/v3.0/ Skowalevskii/assembly/ Saccoglossus_kowalevskii_v3.fasta

Table S2: **List of additional omics data used for the current study.** Latin and trivial names as well as acession numbers are given for data sets that were investigated on top of the NCBI EST and TSA database.

Latin name	Trivial name	GEO accession/ version	Transcriptome source
Callorhinchus milii	ghost shark	GSM643959	http://esharkgenome.imcb.a-star.edu.sg
Leucoraja erinacea	little skate	GSM643957	http://www.skatebase.org/ downloads
Scyliorhinus canicula	catshark	GSM643958	http://www.skatebase.org/ downloads
Gallus gallus	chicken	[20]	http://www.chickest.udel.edu
Taeniopygia guttata	zebra finch	[21]	http://songbirdtranscriptome.net/
		[22]	http://titan.biotec.uiuc.edu/cgi-bin/ESTWebsite/estima_start?seq Set=songbird3

Author details

References

1. Felsenstein, J.: PHYLIP (Phylogeny Inferernce Package): version 3.2 (20.10.2015). http://evolution.genetics.washington.edu/phylip/faq.html Accessed 03.05.2017
2. Kumar, S., Stecher, G., Suleski, M., Hedges, S.B.: TimeTree: A Resource for Timelines, Timetrees, and Divergence Times. Molecular biology and evolution **34**(7), 1812–1819 (2017). doi:10.1093/molbev/msx116
3. Huson, D.H., Scornavacca, C.: Dendroscope 3: an interactive tool for rooted phylogenetic trees and networks. Systematic biology **61**(6), 1061–1067 (2012). doi:10.1093/sysbio/sys06
4. Stover, B.C., Muller, K.F.: TreeGraph 2: combining and visualizing evidence from different phylogenetic analyses. BMC bioinformatics **11**, 7 (2010). doi:10.1186/1471-2105-11-
5. Finn, R.D., Bateman, A., Clements, J., Coggill, P., Eberhardt, R.Y., Eddy, S.R., Heger, A., Hetherington, K., Holm, L., Mistry, J., Sonnhammer, E.L.L., Tate, J., Punta, M.: Pfam: the protein families database. Nucleic acids research **42**(Database issue), 222–30 (2014). doi:10.1093/nar/gkt122
6. Kawano-Yamashita, E., Koyanagi, M., Shichida, Y., Oishi, T., Tamotsu, S., Terakita, A.: beta-arrestin functionally regulates the non-bleaching pigment parapinopsin in lamprey pineal. PloS one **6**(1), 16402 (2011). doi:10.1371/journal.pone.001640
7. Smith, J.J., Baker, C., Eichler, E.E., Amemiya, C.T.: Genetic consequences of programmed genome rearrangement. Current biology : CB **22**(16), 1524–1529 (2012). doi:10.1016/j.cub.2012.06.02
8. Larkin, M.A., Blackshields, G., Brown, N.P., Chenna, R., McGettigan, P.A., McWilliam, H., Valentin, F., Wallace, I.M., Wilm, A., Lopez, R., Thompson, J.D., Gibson, T.J., Higgins, D.G.: Clustal W and Clustal X version 2.0. Bioinformatics (Oxford, England) **23**(21), 2947–2948 (2007). doi:10.1093/bioinformatics/btm40
9. Zhuang, T., Vishnivetskiy, S.A., Gurevich, V.V., Sanders, C.R.: Elucidation of inositol hexaphosphate and heparin interaction sites and conformational changes in arrestin-1 by solution nuclear magnetic resonance. Biochemistry **49**(49), 10473–10485 (2010). doi:10.1021/bi101596
10. Boardman, P.E., Sanz-Ezquerro, J., Overton, I.M., Burt, D.W., Bosch, E., Fong, W.T., Tickle, C., Brown, W.R.A., Wilson, S.A., Hubbard, S.J.: A Comprehensive Collection of Chicken cDNAs. Current Biology **12**(22), 1965–1969 (2002). doi:10.1016/S0960-9822(02)01296-
11. Renninger, S.L., Gesemann, M., Neuhauss, Stephan C F: Cone arrestin confers cone vision of high temporal resolution in zebrafish larvae. The European journal of neuroscience **33**(4), 658–667 (2011). doi:10.1111/j.1460-9568.2010.07574.

12. Rogozin, I.B., Carmel, L., Csuros, M., Koonin, E.V.: Origin and evolution of spliceosomal introns. Biology direct **7**, 11 (2012). doi:10.1186/1745-6150-7-1
13. Flicek, P., Amode, M.R., Barrell, D., Beal, K., Billis, K., Brent, S., Carvalho-Silva, D., Clapham, P., Coates, G., Fitzgerald, S., Gil, L., Girón, C.G., Gordon, L., Hourlier, T., Hunt, S., Johnson, N., Juettemann, T., Kähäri, A.K., Keenan, S., Kulesha, E., Martin, F.J., Maurel, T., McLaren, W.M., Murphy, D.N., Nag, R., Overduin, B., Pignatelli, M., Pritchard, B., Pritchard, E., Riat, H.S., Ruffier, M., Sheppard, D., Taylor, K., Thormann, A., Trevanion, S.J., Vullo, A., Wilder, S.P., Wilson, M., Zadissa, A., Aken, B.L., Birney, E., Cunningham, F., Harrow, J., Herrero, J., Hubbard, Tim J P, Kinsella, R., Muffato, M., Parker, A., Spudich, G., Yates, A., Zerbino, D.R., Searle, Stephen M J: Ensembl 2014. Nucleic acids research **42**(Database issue), 749–55 (2014). doi:10.1093/nar/gkt119
14. Altschul, S.F., Gish, W., Miller, W., Myers, E.W., Lipman, D.J.: Basic local alignment search tool. Journal of molecular biology **215**(3), 403–410 (1990). doi:10.1016/S0022-2836(05)80360-
15. Thibaud-Nissen, F., Souvorov, Alexander Murphy, Terence, DiCuccio, M., Kitts, P.: Eukaryotic Genome Annotation Pipeline, Berthesda (2013). http://www.ncbi.nlm.nih.gov/books/NBK169439/
16. Sterne-Marr, R., Gurevich, V.V., Goldsmith, P., Bodine, R.C., Sanders, C., Donoso, L.A., Benovic, J.L.: Polypeptide variants of beta-arrestin and arrestin3. The Journal of biological chemistry **268**(21), 15640–15648 (1993)
17. Attramadal, H., Arriza, J.L., Aoki, C., Dawson, T.M., Codina, J., Kwatra, M.M., Snyder, S.H., Caron, M.G., Lefkowitz, R.J.: Beta-arrestin2, a novel member of the arrestin/beta-arrestin gene family. The Journal of biological chemistry **267**(25), 17882–17890 (1992)
18. Komori, N., Cain, S.D., Roch, J.M., Miller, K.E., Matsumoto, H.: Differential expression of alternative splice variants of beta-arrestin-1 and -2 in rat central nervous system and peripheral tissues. The European journal of neuroscience **10**(8), 2607–2616 (1998)
19. Rapoport, B., Kaufman, K.D., Chazenbalk, G.D.: Cloning of a member of the arrestin family from a human thyroid cDNA library. Molecular and cellular endocrinology **84**(3), 39–43 (1992)
20. Carre, W., Wang, X., Porter, T.E., Nys, Y., Tang, J., Bernberg, E., Morgan, R., Burnside, J., Aggrey, S.E., Simon, J., Cogburn, L.A.: Chicken genomics resource: sequencing and annotation of 35,407 ESTs from single and multiple tissue cDNA libraries and CAP3 assembly of a chicken gene index. Physiological genomics **25**(3), 514–524 (2006). doi:10.1152/physiolgenomics.00207.200
21. Jarvis, E.D., Smith, V.A., Wada, K., Rivas, M.V., McElroy, M., Smulders, T.V., Carninci, P., Hayashizaki, Y., Dietrich, F., Wu, X., McConnell, P., Yu, J., Wang, P.P., Hartemink, A.J., Lin, S.: A framework for integrating the songbird brain. Journal of comparative physiology. A, Neuroethology, sensory, neural, and behavioral physiology **188**(11-12), 961–980 (2002). doi:10.1007/s00359-002-0358-
22. Replogle, K., Arnold, A.P., Ball, G.F., Band, M., Bensch, S., Brenowitz, E.A., Dong, S., Drnevich, J., Ferris, M., George, J.M., Gong, G., Hasselquist, D., Hernandez, A.G., Kim, R., Lewin, H.A., Liu, L., Lovell, P.V., Mello, C.V., Naurin, S., Rodriguez-Zas, S., Thimmapuram, J., Wade, J., Clayton, D.F.: The Songbird Neurogenomics (SoNG) Initiative: community-based tools and strategies for study of brain gene function and evolution. BMC genomics **9**, 131 (2008). doi:10.1186/1471-2164-9-13
